# Development of a gut microbiota model for the analysis of bacterial modifications of xenobiotics

**DOI:** 10.1007/s00253-026-13861-7

**Published:** 2026-05-30

**Authors:** Natalie Hager, Jan-Lorenz Weyers, Laura Falk, Maike Passon, Marie-Christine Simon, Waldemar Seel, Jean-Lou Christian Michel Dorne, Uwe Deppenmeier

**Affiliations:** 1 Institute of Microbiology and Biotechnology, University of Bonn, Meckenheimer Allee 168, 53115 Bonn, Germany; 2 Institute for Food Chemistry, University of Bonn, Friedrich-Hirzebruch-Allee 7, 53115 Bonn, Germany; 3 Institute for Nutrition and Microbiota, University of Bonn, Katzenburgweg 7, 53115 Bonn, Germany; 4 Institute for Human Genetics, NGS Core Facility, University Hospital Bonn, Venusberg-Campus 1, 53127 Bonn, Germany; 5 Methodology and Scientific Support Unit, European Food Safety Authority (EFSA), Via Carlo Magno 1A, 43126 Parma, Italy

**Keywords:** Gut microbiota, Xenobiotics, In vitro colon model, Toxicomicrobiomics, Drug metabolism, Physiologically based kinetic modeling (PBK)

## Abstract

**Abstract:**

The human gut microbiota influences host physiology by metabolizing xenobiotics, such as drugs, dietary additives, and environmental contaminants. To enable standardized assessment of microbial xenobiotic metabolism, we established a defined bacterial colon model from pooled human fecal samples, by preparing clean bacterial cell suspensions using density gradient centrifugation. The bacterial cell fraction remained structurally intact, allowing long-term preservation at −70 °C with glycerol as a cryoprotectant. The bacteria were fully reactivated without compromising cellular integrity. Glycerol as a disturbing substrate was removed by washing steps. The system preserved microbial diversity, enzymatic activity, and metabolic functionality over 24 h under anaerobic conditions. Unlike fecal-based models, the model was free from non-cellular contaminants, which could cause unpredictable interactions between test compounds and reactive components within the fecal matrix. Enzymatic assays demonstrated hydrolytic, reductive, and proteolytic activities comparable to native feces. 16S rRNA gene sequencing confirmed taxonomic stability and compositional shifts in response to different substrates. A fiber-rich substrate proved to be optimal for maintaining the bacterial composition over 24 h. We further applied the model to investigate the microbial biotransformation of selected model xenobiotics using high-performance liquid chromatography and mass spectrometry, capturing substrate-specific conversion patterns and formation of expected primary products. A versatile addition of food ingredients, dietary supplements, pharmaceuticals, and environmental chemicals is possible to analyze their effects on the composition of the microbiota, its enzymatic activity, and the excretion of metabolites and end products. Together, this bacterial colon model provides a reproducible, high-throughput capable and ethically viable platform for pharmaco microbiomic studies and next-generation risk assessment.

**Key points:**

- *A novel bacterial colon model preserves human gut microbial diversity and activity.*
- *Fiber-rich substrates restore enzymatic function and SCFA production.*
- *The model enables standardized screening of microbial xenobiotic metabolism.*

**Supplementary Information:**

The online version contains supplementary material available at 10.1007/s00253-026-13861-7.

## Introduction

The human body serves as a favored environment for a vast diversity of bacteria, archaea, and fungi (Schmidt et al. 2018). Collectively referred to as the human microflora or gut microbiota, these microorganisms inhabit various body sites and typically coexist with their host in a mutualistic relationship. In an adult human, the body harbors up to 10^14^ prokaryotic cells (Sender et al. 2016), with the majority residing in the gastrointestinal tract. This complex microbial population interacts actively with the gut lymphoid tissues and the immune system of the host (Gensollen et al. 2016). Furthermore, the gut microbiota plays a pivotal role in maintaining intestinal health and influencing the development of both intestinal and non-intestinal diseases. A major class of non-host-derived substances is xenobiotics, a diverse group of chemicals including pharmaceuticals, environmental pollutants, food additives, and naturally occurring compounds that may exert toxic effects at elevated concentrations (Soucek 2011). Given their structural diversity and widespread presence, the estimated interaction of more than 25,000 different compounds with the gut microbiota underscores its critical function in shaping host responses (Lindell et al. 2022). Unlike human metabolism, which primarily relies on hepatic oxidation and conjugation, the gut microbiome harbors more than 3.3 million genes exceeding the human genome by over 150-fold (Qin et al. 2010). Hence, the gut microbiome provides a remarkably broad enzymatic repertoire, including hydrolysis, reduction, and deconjugation reactions (Zimmermann et al. 2019). These microbial transformations can significantly impact the half-life, bioavailability, and pharmacokinetics of xenobiotics by altering their toxicity, metabolite formation, or pharmacological effects (Koppel et al. 2017). Prominent examples include the activation of the ulcerative colitis drug sulfasalazine through azo bond cleavage (Lima et al. 2024), the deglucuronidation of non-steroidal anti-inflammatory drugs such as diclofenac (Boelsterli et al. 2013), the acetylation-mediated inactivation of 5-aminosalicylic acid (Deloménie et al. 2001), as well as the modulation of drug efficacy and toxicity through reduction, hydrolysis, and competition with host metabolic pathways, as observed for digoxin (Haiser et al. 2013), irinotecan (Takasuna et al. 1996), and acetaminophen (Clayton et al. 2009). By shaping the chemical landscape of the intestinal lumen, the gut microbiota affects not only systemic exposure but also presents both challenges and opportunities for contemporary chemical risk assessment. Traditional approaches, based on animal studies, clinical trials, and epidemiological data, often neglect the role of microbial metabolism in modulating xenobiotic fate (Stevanoska et al. 2024). In response, emerging fields such as toxicomicrobiomics aim to elucidate the bidirectional interactions between gut microbes and toxicants (Abdelsalam et al. 2020). Incorporating microbiome dynamics into physiologically based kinetic models (PBK models) has the potential to improve the accuracy of exposure and risk predictions. This underscores the need for advanced gut microbiome models that more accurately reflect human microbial diversity and functional capacity. Such standardized systems are not only critical for mechanistic understanding but also hold promise for improving preclinical screening and regulatory safety assessment of xenobiotics. Extensive efforts have been made to create experimental in vitro and ex vivo models of the human intestine, enabling the analysis of gastrointestinal pathophysiology and the intricate involvement of bacteria in these processes (Stevanoska et al. 2024). However, for certain applications, such as screening, standardization, and integration into higher-throughput pipelines, simpler and more accessible models are essential. To address this need, we developed a streamlined and defined in vitro bacterial colon model optimized for reproducibility, ease of use, and compatibility with routine xenobiotic screening workflows. Together, these features make the system particularly suitable for rapid, high-throughput applications in toxicomicrobiomics, including studies on how xenobiotics are transformed by the gut microbiota.

## Material and methods

### Chemicals

All reagents, chemicals, and substrates were obtained from commercial suppliers including Carl Roth GmbH & Co. KG (Karlsruhe, Germany), Sigma-Aldrich (Darmstadt, Germany), as well as Tokyo Chemical Industry Co., Ltd. (Tokyo, Japan), BLDpharm Co., Ltd. (Shanghai, China), Chemos GmbH & Co. KG (Regenstauf, Germany), and Cayman Chemical Company (Ann Arbor, MI, USA). The gases CO_2_ (99.9%), H_2_ (99.9%), and N_2_ (99.9%) were obtained from Air Liquide (Düsseldorf, Germany). All substances were of analytical grade or higher and were used without further purification.

### Study design

The objective of this study was to develop and validate a new and standardized in vitro colon model based on bacterial communities isolated from human feces. The primary objective was to preserve the taxonomic diversity and metabolic functionality of the gut microbiota while enabling controlled and reproducible functional assays. Diluted fecal samples from healthy individuals were used to obtain both untreated reference inocula and standardized bacterial preparations for the colon model. To minimize interindividual variability, donors were randomly assigned to groups of three, and their samples were pooled under anaerobic conditions, allowing for randomization at both the collection and application stages.

### Sample collection

Fecal samples were obtained from 20 healthy male and female volunteers aged 20 to 60 years who had not received antibiotic treatment within the 6 months prior to recruitment. The study protocol was approved by the Ethics Committee of the University of Bonn and is registered in the German Clinical Trials Register (registration number DRKS00036882, registered on 26 September 2025; https://drks.de/search/de/trial/DRKS00036882), and all procedures were conducted in accordance with the relevant guidelines and regulations. Each participant collected two fecal samples using a standardized collection kit. Immediately after collection, 5 g of fresh stool was diluted in 50 mL of pre-anaerobized modified SHIME® saline buffer (29.8 mM NaHCO_3_, 34.2 mM NaCl, 18.7 mM NH_4_Cl, 49.5 mM K_2_HPO_4_, 49.8 mM KH_2_PO_4_, 0.07 mM CaCl_2_ × 2 H_2_O, 0.03 mM MgSO_4_ × 7 H_2_O, pH 7.0), which had been flushed with N_2_/CO_2_ (80%/20%) and sealed in serum flasks with butyl rubber stoppers (Van De Wiele et al. 2015). Following sample addition, the flasks were flushed twice with 50 mL of 80% N_2_ and 20% CO_2_ to remove the air in the headspace. The samples were stored at 4 °C until further processing (< 2 h).

### Sample processing for the* in vitro* bacterial colon model

Our model was based on the isolation of intact bacterial communities from human feces. Bacterial isolation was performed using a modified density gradient centrifugation protocol first described by Hevia et al. (2015). To detach bacteria adhering to particulate matter and embedded within fecal biofilms, 0.001% (w/v) cetyltrimethylammonium bromide (CTAB) was added to each diluted fecal sample 30 min prior to further processing (Macfarlane et al. 1997). All steps involving open sample handling were conducted under strictly anaerobic conditions (79% N_2_, 19% CO_2_, 2% H_2_) within a vinyl anaerobic chamber (Coy Laboratory Products, Grass Lake, Michigan, USA). Samples from three donors were randomly pooled for each preparation to reduce variability among individuals. From each pooled and diluted fecal suspension, 15 mL were layered onto 3.5 mL of 55% (w/v) Nycodenz® (Serumwerk Bernburg AG, Bernburg, Germany) in anaerobic centrifuge tubes. The samples were then centrifuged at 4000 × g for 30 min at room temperature. Following centrifugation, three distinct layers were obtained: an upper layer containing extracellular enzymes and soluble components, an intermediate opaque layer composed of bacterial cells, and a lower layer consisting of insoluble debris.

To further purify the extracellular enzyme (exoenzyme) fraction, the supernatant was centrifuged at 18,000 × g for 3 min at 4 °C. The resulting preparation was then sterile-filtered three times through 0.22-µm syringe filters to ensure complete removal of remaining microbial cells. The sterile exoenzyme fraction was stored anaerobically at −70 °C until further use. The middle layer, which contained the gut bacteria, was collected and referred to as the bacterial colon model. The isolated microbiota was then diluted in sterile 50% (v/v) glycerol and stored at −70 °C under anaerobic conditions. Prior to use in experiments, the cryopreserved cells were washed three times with modified SHIME® saline (18,000 × g, 3 min, 4 °C) to remove any remaining Nycodenz®, glycerol, and particulate material. The final cell suspension was adjusted to an optical density at 600 nm (OD_600_) of 10, corresponding to approximately 1.43 × 10^10^ cells mL^−1^, as determined microscopically using a Neubauer counting chamber (Hager et al. 2025). This microbial density was about 16% of the mean in vivo bacterial concentration in the human colon, estimated at 8.8 × 10^10^ cells mL^−1^ (assuming a fecal density of 1.04 g mL^−1^ (Sender et al. 2016)). The system can be upscaled to achieve cell numbers corresponding to up to 50% of the in vivo abundance of colonic bacteria.

Since this washing procedure eliminates all extracellular enzymes, a targeted enzymatic regeneration step was necessary to restore the microbial metabolic functionality. Therefore, distinct substrate formulations were tested for their ability to support functional recovery while preserving taxonomic integrity and metabolic activity. All regeneration media were based on a modified SHIME® salt solution serving as a basal medium to approximate intestinal luminal conditions. Four substrate conditions were assessed: (i) a no-substrate control, (ii) a fiber-rich medium, (iii) a Western-style medium supplemented with bile salts, and (iv) the corresponding Western-style medium without bile salts. The detailed composition of all media is summarized in Table S1. In brief, the fiber-rich formulation was designed to mimic a polysaccharide-rich colonic environment, whereas the Western-style formulations represented a nutrient milieu richer in starch, protein, and fatty acids, with the bile salt-supplemented variant additionally accounting for bile exposure under high-fat dietary conditions. Due to the limited purity of commercially available polysaccharides, xylan, mucin, and pectin were purified by triple ethanol precipitation (100 mL of a 10% solution in 900 mL 99.9% ethanol), followed by lyophilization and purity verification by HPLC. All regeneration incubations were performed for 24 h at 37 °C under strictly anaerobic conditions.

### Microscopic imaging

To evaluate the morphological characteristics of the isolated bacterial colon model, the samples were examined using bright-field microscopy. To immobilize the cells, 1.5% (w/v) low-melting-point agarose was mixed with SHIME® saline and melted at 70 °C. The melted agarose was dispensed onto concave microscope slides (Thermo Fisher Scientific, Waltham, MA, USA) and covered with a coverslip to allow solidification. Washed bacterial suspensions (2–5 μL) were then applied to the agarose surface and covered with a coverslip. Images were acquired using a Nikon Ti2-E inverted microscope equipped with a 160× oil-immersion objective and a Prime BSI sCMOS camera (Teledyne Photometrics, Tucson, AZ, USA; pixel size 107 nm).

### 16S rRNA amplicon sequencing for microbial community profiling

Microbial community profiling was performed by 16S rRNA gene amplicon sequencing using the Illumina MiSeq platform (Illumina Inc., San Diego, CA, USA). Total genomic DNA was extracted from 150 mg fecal material or 100 µL of microbial cell pellet (1 × 10^9^ cells) using the Quick-DNA™ Fecal/Soil Microbe Microprep Kit (Zymo Research, Irvine, CA, USA), following the manufacturer’s instructions. Mechanical lysis was performed via bead beating on a vortex mixer equipped with a horizontal adapter. DNA was eluted in a final volume of 50 µL. Concentrations were measured using a BioSpectrometer® (Eppendorf, Hamburg, Germany), and stored at −20 °C until further analysis.

Amplicon libraries targeting the V3–V4 region of the 16S rRNA gene were generated using the primer pair Bakt_341F (5′-CCTACGGGNGGCWGCAG-3′) and Bakt_805R (5′-GACTACHVGGGTATCTAATCC-3′). PCR amplification was carried out using 2×KAPA HiFi HotStart ReadyMix polymerase (Roche, Mannheim, Germany) under the following cycling conditions: 22 cycles of 95 °C (denaturation), 55 °C (annealing), and 72 °C (extension), followed by a final elongation step.

To attach Illumina Nextera XT adapter sequences, a second PCR was performed using indexed primers. Amplicons were purified using AMPure XP beads (Beckman Coulter, Krefeld, Germany), and quantified by the Qubit™ dsDNA HS Assay Kit (Thermo Fisher Scientific, Waltham, MA, USA), as well as normalized and pooled manually. The pooled libraries were denatured in 0.2 N NaOH, diluted to 10 pM, and spiked with 20% PhiX control DNA (Illumina, San Diego, CA, USA) prior to sequencing. Sequencing was performed on an Illumina MiSeq platform using the MiSeq Reagent Kit v3 with 2 × 300 bp paired-end chemistry. Raw sequence reads were demultiplexed using MiSeq Reporter v2.5 and further processed with the QIIME 2 pipeline (version 2021.4). Quality control and denoising were performed using the DADA2 plugin (Callahan et al. 2016), applying a minimum quality threshold of Q30. Forward and reverse reads were truncated at 270 bp and 210 bp, respectively, to remove low-quality regions, and chimeric sequences were filtered out subsequently.

### Measurement of enzyme activities

Microbial enzyme activities were determined by spectrophotometric assays conducted under standardized conditions using a Jasco V-600 UV/Vis spectrophotometer (Jasco, Groß-Umstadt, Germany). All reactions were carried out in 100 mM potassium phosphate buffer (pH 7.0) supplemented with 0.5 mM CaCl_2_ and MgCl_2_.

To measure intracellular dehydrogenase activities, bacterial cells were lysed by bead beating. Therefore, 1 mL of the bacterial suspension in modified SHIME® saline was supplemented with 0.1 mM EDTA, 0.1% (v/v) Triton X-100, and a 1× protease inhibitor cocktail (Sigma-Aldrich, Darmstadt, Germany). Bead beating was performed on a vortex mixer equipped with a horizontal adapter for 15 min at 4 °C, followed by centrifugation at 17,000 × g for 5 min at 4 °C. The supernatant containing the crude enzyme extract was collected and used directly for enzyme assays. Lactate dehydrogenase (lactate DH) and malate dehydrogenase (malate DH) activities were determined by monitoring nicotinamide adenine dinucleotide (NADH) consumption at 340 nm (*ε* = 6.22 mM⁻^1^ cm⁻^1^) at 37 °C. For lactate DH, 10–50 µL of cell lysate were added to 950–990 µL of 50 mM Tris–HCl buffer (pH 7.4) containing 0.5 mM pyruvate and 0.25 mM NADH. Malate DH activity was measured under identical conditions using 0.5 mM oxaloacetate and 0.25 mM NADH as substrates.

Extracellular enzyme activities were determined using colorimetric and dye-linked assays based on the hydrolysis of p-nitrophenyl (pNp), Remazol Brilliant Blue (RBB)-, and Azo-conjugated substrates. Esterase and glycosidase activities were measured by the enzymatic cleavage of pNp substrates (acetate, butyrate, palmitate, α-glucopyranoside, β-galactopyranoside). A total of 10–75 µL of sample were incubated with 2–4 µL of the respective pNp substrate (125–250 mM in DMSO, ethanol, or CH₂Cl₂) in buffer to a final reaction volume of 500 µL. After 10–15 min at 37 °C, reactions were stopped with 500 µL ethanol and centrifuged (18,000 × g, 5 min). Absorbance was measured at 410 nm (*ε* = 18.3 mM^−1^ cm^−1^). Xylanase and cellulase activities were determined using RBB-xylan (2.5 mg mL^−1^), Azo-xylan (6.25 mg mL^−1^), and Azo-cellulose (6.25 mg mL^−1^). Samples (50–100 µL) were incubated with 100 µL substrate in a final volume of 500 µL at 37 °C for 2 h. Reactions were stopped with 1 mL precipitation buffer (10 g sodium acetate × 3 H_2_O, 1 g zinc acetate in 50 mL H_2_O, pH 5.0, containing 200 mL ethanol) and centrifuged (18,000 × g, 5 min). Absorbance was measured at 595 nm (*ε* = 6.17 mM^−1^ cm^−1^). Amylase activity was determined using 100 µL Red-starch (20 mg mL^−1^ in 0.5 M KCl) incubated with 10–20 µL sample and 180–200 µL buffer for 20 min at 37 °C. Reactions were stopped with 500 µL ethanol, centrifuged (18,000 × g, 5 min), and absorbance was measured at 510 nm (*ε* = 6.17 mM^−1^ cm^−1^). Protease activity was assayed using 100 µL Azo-casein (20 mg mL^−1^) incubated with 10–20 µL sample and 80–100 µL buffer for 10 min at 37 °C. Reactions were terminated with 600 µL 5% (w/v) trichloroacetic acid (TCA), centrifuged (18,000 × g, 5 min), and absorbance was recorded at 440 nm. Enzymatic activity was calculated using absorbance values to determine the reaction rate (∆E min^−1^), which was converted to µmol min^−1^ using the Beer-Lambert law and the appropriate molar extinction coefficient. Specific activity was expressed as nmol min^−1^ mg protein^−1^. Protein concentrations were measured at 595 nm using the Bradford assay with bovine serum albumin (BSA) as standard (Bradford 1976).

### SCFA quantification using HPLC

The short-chain fatty acids (SCFAs) were quantified using a high-performance liquid chromatography (HPLC) system equipped with refractive index (RI) and ultraviolet (UV) detectors. Separation was achieved on an Aminex HPX-87H column (300 mm × 7.8 mm, Bio-Rad, Munich, Germany) at 65 °C with 5 mM sulfuric acid (H_2_SO_4_) as the mobile phase at a flow rate of 0.6 mL min^−1^. Prior to injection, fecal and colon model samples were clarified by mixing 100 µL of sample with 50 µL Carrez I solution (0.15 g mL^−1^ K_4_[Fe(CN)_6_]) and 50 µL Carrez II solution (0.30 mg mL^−1^ ZnSO_4_ x 7 H_2_O). The mixture was vortexed and centrifuged at 18,000 × g for 2 min. Subsequently, 70 µL of the supernatant were diluted with 140 µL of 5 mM H_2_SO_4_. An additional centrifugation step (18,000 × g, 2 min) was performed to remove any remaining particulates. Quantification was performed using external calibration curves.

### Xenobiotic assays quantified with HPLC and supported with LC–MS

To evaluate microbial xenobiotic metabolism under defined conditions, incubations were performed with model substrates representing key biotransformation classes with roxatidine-acetate, 4-acetoxyacetanilide (ester hydrolysis), sulindac (sulfoxide reduction), sulfasalazine (azo bond reduction), as well as nitrendipine and chloramphenicol (nitro group reduction). Quantification of precursor compounds and metabolites was carried out by HPLC, and results were validated by liquid chromatography-mass spectrometry (LC–MS). To enable parameter-based comparison of compound conversion across incubation matrices, apparent first-order kinetic parameters were calculated from precursor compound depletion data. Only quantifiable time points were considered. Because these values represent apparent kinetic estimates derived from a limited number of sampling points, they were interpreted primarily for comparative purposes. Detailed experimental conditions and analytical parameters are provided in Supplementary Tab. S2.

### Statistics

All statistical analyses were performed using GraphPad Prism (version 8.0.2, GraphPad Software, San Diego, CA, USA). For taxonomic profiling, changes in microbial composition at the genus level were assessed by comparing relative abundances between baseline (T0) and post-incubation (T24) under four different substrate conditions. Dominant genera (≥ 1% mean relative abundance) and low-abundance genera (≤ 1%) were visualized using heatmaps and bar plots. For each genus, the condition showing the smallest deviation was identified. Differences across conditions were evaluated using Kruskal–Wallis tests, and conditions with *p* < 0.05 were considered statistically significant. Enzymatic activity measurements were expressed as specific activity (nmol min^−1^ mg protein^−1^), normalized to protein content. Comparisons between original fecal samples and colon model compartments were analyzed using paired two-tailed *t*-tests (*n* = 8–10). For functional dynamics during substrate incubation, enzymatic activities at T24 were compared to T0 values (baseline set to 100%) using paired *t*-tests (*n* = 8–10). The SCFA concentrations (acetate, propionate, butyrate) were quantified by HPLC. Data were presented as mean ± standard deviation (SD), with technical replicates (*n* = 8) for controls and pooled replicates (*n* = 17) for substrate-supplemented samples. Comparisons between fecal and colon model samples, with or without substrate supplementation, were performed using unpaired *t*-tests. Significance levels were defined as follows: *p ≤ 0.05, **p ≤ 0.01, ***p ≤ 0.001, ****p ≤ 0.0001. More details are described in the supplementary material.

## Results

### Development of an in vitro model reflecting the natural microbiota in the human colon

**Fig. 1:**
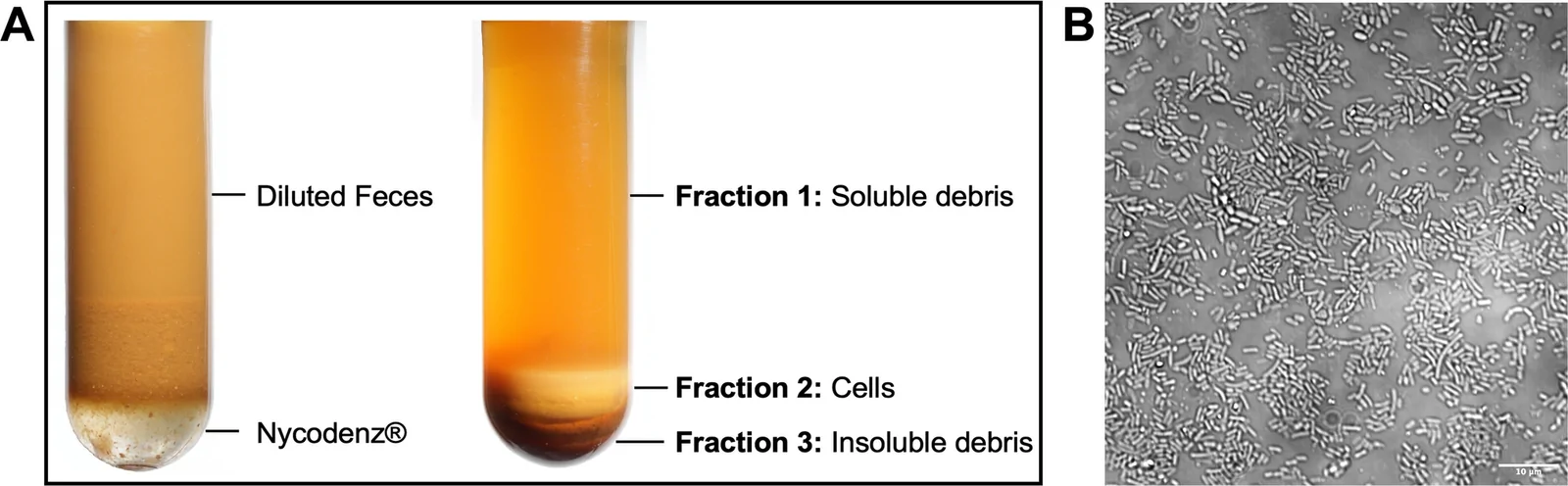
Separation of the bacterial colon model from human fecal samples. **A** Schematic representation of the density-gradient centrifugation. The left panel shows diluted fecal samples layered on the density-gradient medium, and the right panel illustrates the separated fractions. **B** Representative microscopic images of the isolated bacterial cell fraction (1,600 × magnification).

To establish a physiologically relevant in vitro model, particularly suited for investigating the contribution of the gut microbiota to xenobiotic transformations, a new method for the isolation and functional regeneration of native microbial communities from human fecal samples was developed and validated. Fecal samples from multiple donors were diluted 1:10 (w/v) and pooled to minimize interindividual variability. CTAB (0.001% w/v) was added as a mild detergent to detach bacteria from particle surfaces and biofilm structures (Hevia et al. 2015). This was followed by a Nycodenz® density-gradient centrifugation (Macfarlane et al. 1997), which separated the fecal microbiota from stool samples. Three fractions were obtained (Fig. 1A): (i) an upper aqueous layer enriched in extracellular components, including secreted enzymes and low-density non-cellular material such as mucosal residues, lipids, and proteins; (ii) an intermediate opaque layer containing intact bacterial cells representative of the phylogenetic diversity of the human colonic microbiota, hereafter referred to as the bacterial colon model; and (iii) a lower dense fraction containing undigested dietary fibers, host-derived debris, and cell fragments. To assess the efficiency of bacterial cell isolation, total protein concentrations were determined in the original fecal slurry, the purified colon model fraction, and the corresponding supernatant after centrifugation. The isolation procedure achieved an average recovery rate of 86 ± 17% relative to the initial protein content, indicating efficient preservation of microbial biomass. Amplicon sequencing of native feces and the corresponding colon model further showed that Nycodenz® purification did not significantly alter the overall microbial community composition (Supplementary Fig. S1). No significant differences were observed in alpha-diversity metrics, including observed features, Shannon diversity, Pielou’s evenness, and Faith’s phylogenetic diversity. Beta-diversity analyses based on Bray–Curtis, Jaccard, weighted UniFrac, and unweighted UniFrac distances did not reveal a clear separation between both sample types. Genus-level abundance profiles were also highly comparable after purification, indicating that the overall community structure was largely retained. Thus, the protocol enabled a clean and targeted isolation of the intestinal microbial community, minimizing matrix-derived interference and ensuring high compatibility with downstream functional, biochemical, and analytical assays. Bright-field microscopy revealed a morphologically diverse and densely populated bacterial community exhibiting high levels of motility and structural heterogeneity (Fig. 1B). Bacterial cells showed distinct morphotypes, indicating a broad phylogenetic spectrum. Importantly, the preparation was free of visible particulate contamination or residual matrix components, which could otherwise interfere with subsequent analyses.

The bacterial cell fraction remained structurally intact, allowing long-term preservation at −70 °C using glycerol as a cryoprotectant, followed by successful reactivation without compromising cellular integrity. For reactivation, samples were thawed and washed twice to remove glycerol, which would otherwise serve as a carbon source for certain bacteria and thereby alter both the metabolic output and microbial community composition (De Weirdt et al. 2010). The presence of glycerol indeed affected the microbial community structure, as reflected by a shift in the SCFA profile, characterized by reduced acetate formation and increased levels of propionate, butyrate, and additional, yet unidentified, SCFAs (data not shown).

## Substrate-induced microbial community changes during regeneration

Processing of the bacterial colon model resulted in the loss of extracellular enzymes. These enzymes are not only involved in the degradation of structurally complex dietary polysaccharides such as pectin and xylan (Yüksel et al. 2025) but also play a key role in the microbial biotransformation of xenobiotics that are typically not internalized but undergo extracellular modification (Smacchi and Gobbetti 2000). Restoring this enzymatic activity while maintaining the native community structure was therefore essential for conducting physiologically relevant metabolic studies. To achieve enzymatic regeneration, colon model preparations were incubated anaerobically for 24 h under four defined conditions to evaluate the influence of nutrient composition on microbiota recovery: in the absence of substrates, in the presence of a fiber-rich medium consisting of 1 g L^−1^ each of mucin, pectin, and xylan, and with a Western-style diet formulation, either with or without the addition of bile salts. The fiber-rich substrates contained structurally diverse dietary fibers, designed to mimic fiber-rich nutritional input. In contrast, the Western-style substrate formulation incorporated starch, proteinaceous compounds, a mixture of saturated and unsaturated fatty acids, as well as SCFAs. To assess the effect of host-derived components, bile salts were either included (0.5 g L^−1^) or omitted from the formulation.

**Fig. 2:**
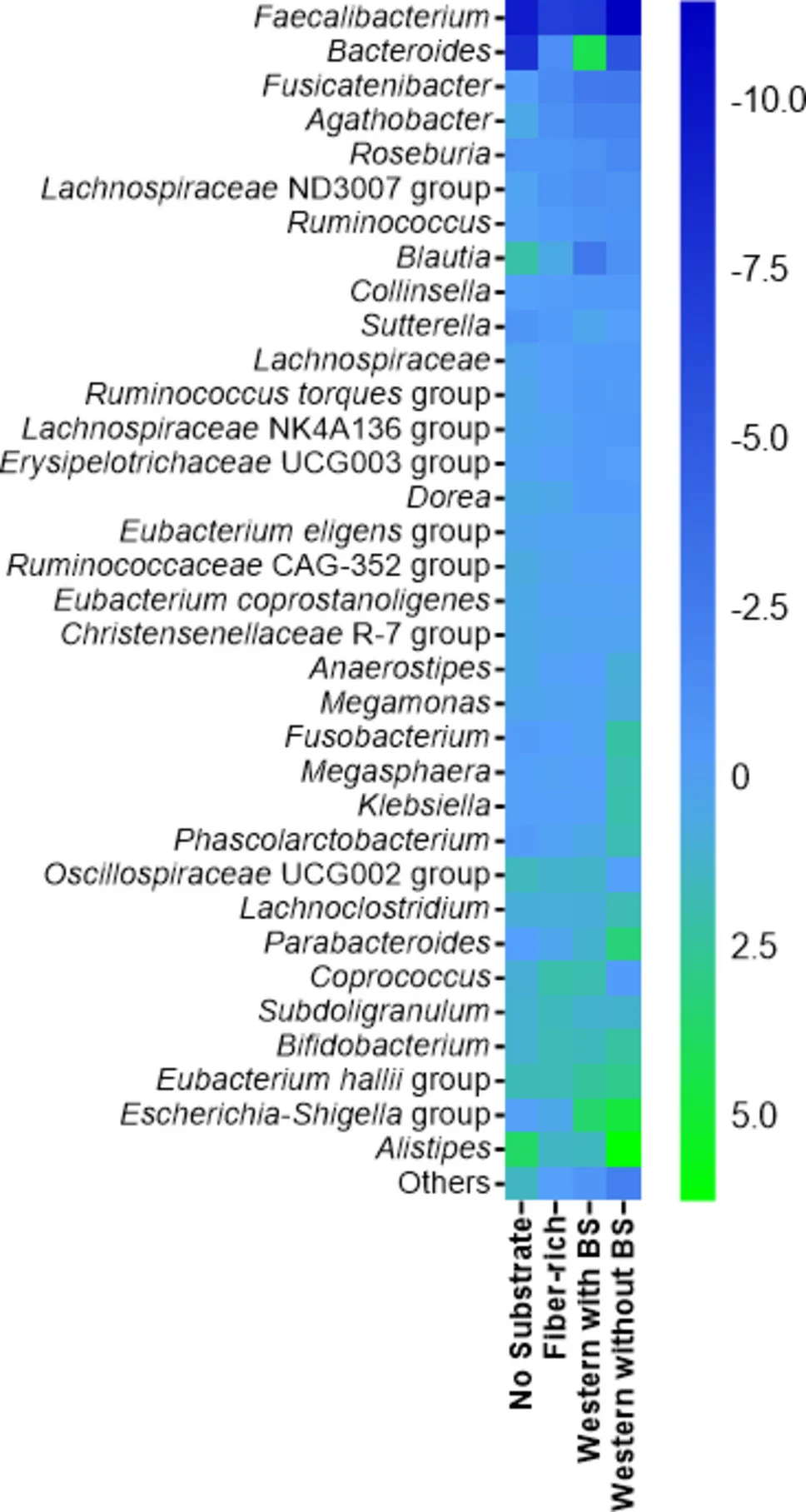
Taxonomic shifts in the gut microbiota composition under different substrate conditions. Heatmap showing changes in the relative abundance of dominant bacterial genera (≥ 1% mean abundance) following 24 h incubation under four substrate conditions: no substrate, fiber-rich medium, Western-style medium supplemented with bile salts (BS), and Western-style medium without bile salts (BS). Values were compared to the initial baseline (T0) as determined by 16S rRNA gene amplicon sequencing

Microbial composition was analyzed at baseline and after incubation using 16S rRNA gene amplicon sequencing. In total, 74 families, 203 genera, and 412 species were identified across all samples, revealing compositional shifts that reflected substrate-dependent selection pressures. For comparative visualization, only genera with a mean relative abundance of > 1% across all conditions (*n* = 34) were included in the heatmap analysis (Fig. 2). All remaining low-abundance genera were grouped into a collective category termed “others.”. The complete list of detected organisms and their substrate-dependent abundances is provided in Supplementary Tab. S3.

Several low-abundance genera (< 5%), such as *Collinsella*, *Dorea*, and members of the Christensenellaceae or Erysipelotrichaceae families, remained stable across all substrate conditions. In contrast, pronounced shifts were observed among dominant taxa, particularly within the genera *Faecalibacterium* and *Bacteroides*, which together represented more than 32% of the initial community (15.4 ± 0.5% and 17.1 ± 0.9%). *Faecalibacterium* showed the strongest decline under Western-style conditions with bile salts, decreasing from 11.5 ± 0.4% to 3.6 ± 0.2% after 24 h. A comparable reduction occurred under substrate-free conditions, with a final abundance of only 5.2 ± 0.2%, emphasizing the sensitivity of this genus to nutrient limitation and substrate composition. In contrast, the fiber-rich medium minimized these losses, preserving a post-incubation abundance of 8.5 ± 0.1%, representing the most effective recovery among all tested conditions. With an initial abundance of 17.1 ± 0.9%, the genus *Bacteroides* also underwent a significant decrease under substrate-deprived conditions, reaching 9.1 ± 0.1%. Species of this genus were also strongly affected by the presence of bile salts under Western-style conditions (5.5 ± 1.5%), whereas the fiber-rich medium sustained an abundance of 15.6 ± 1.3% after 24 h.

**Fig. 3:**
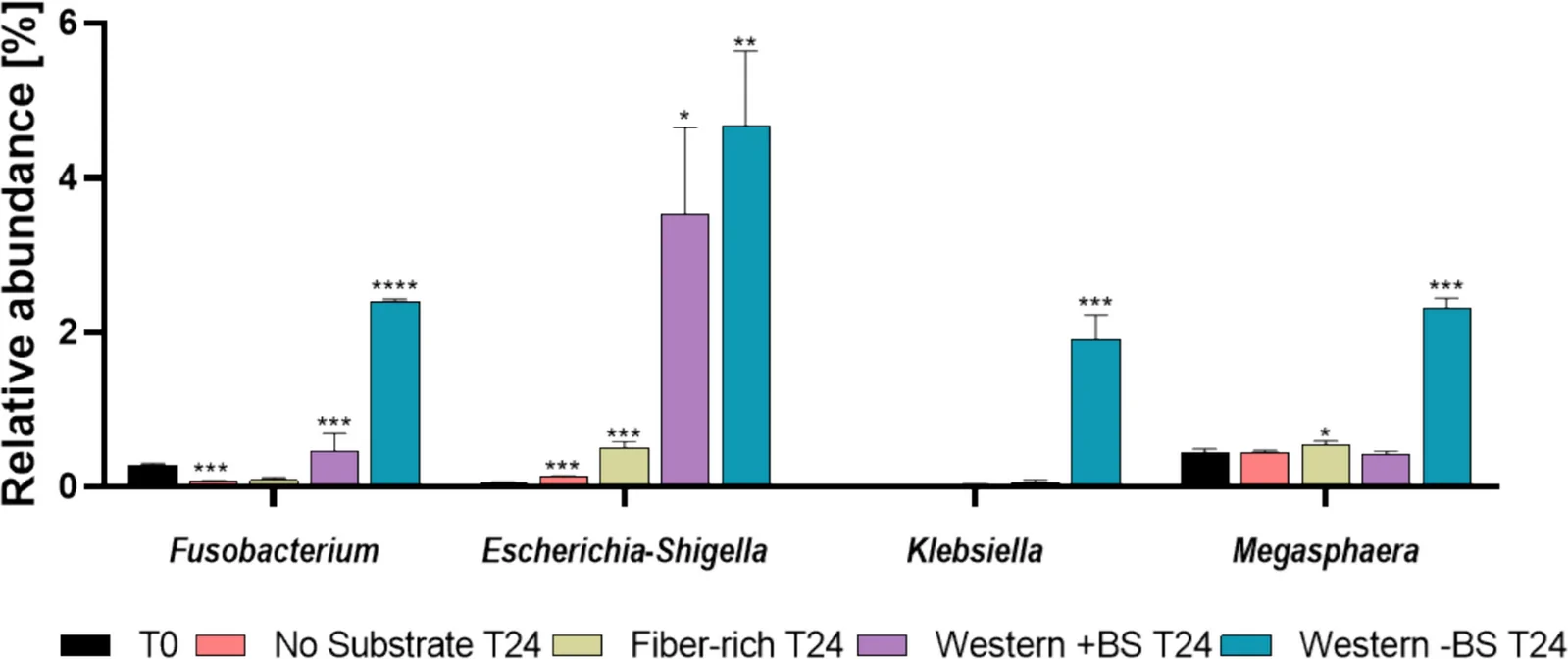
Relative shifts among low-abundance bacterial genera under different substrate conditions. Bar charts illustrate the relative abundance of selected taxa after 24 h incubation under four substrate conditions: no substrate, fiber-rich medium, Western-style medium with bile salts (BS), and Western-style medium without bile salts (BS). Values are shown relative to the initial baseline (T0) as determined by 16S rRNA gene amplicon sequencing. Statistical significance was assessed using unpaired two-tailed *t*-tests (*n* = 3 per condition). Asterisks denote significance levels: *p ≤ 0.05, **p ≤ 0.01, ***p ≤ 0.001, ****p ≤ 0.0001

Additionally, the fiber-rich medium effectively mitigated the loss of key anaerobic commensals such as *Agathobacter*, *Roseburia,* and *Fusicatenibacter*, while supporting the maintenance of several health-associated taxa. These included *Subdoligranulum* (4.7 ± 0.3%), *Bifidobacterium* (3.4 ± 0.3%), and the *Eubacterium hallii* group (4.2 ± 0.1%), which are all known producers of SCFAs implicated in gut homeostasis. The *Escherichia–Shigella* group, typically detected at only 0.05 ± 0.01% at baseline, expanded nearly 100-fold to 4.6 ± 1.0% in the Western-style medium, representing the strongest increase among all taxa (Fig. 3). Under these conditions, *Fusobacterium* and *Klebsiella*, both associated with inflammation and epithelial barrier dysfunction, also showed marked increases to 2.1 ± 0.03% and 1.9 ± 0.3%, respectively (Fig. 3). These findings are consistent with previous reports linking Western dietary patterns to microbial imbalance and pro-inflammatory host responses (Statovci et al. 2017; Christ et al. 2019). *Megasphaera* also expanded notably (1.9 ± 0.1%), potentially reflecting its ability to thrive under altered nutrient availability and reduced competition from strict anaerobes. Detailed statistical evaluations are provided in the Supplementary Material S3. Compared to the Western diet, the changes in the frequency of the genera mentioned were much smaller in the fiber-rich medium or no significant changes in abundance were found. Overall, the fiber-rich medium consistently resulted in the lowest average deviations across genera and preserved the initial community structure the best.

### Analysis of enzymatic activity and fermentative capacity in the bacterial colon model

**Fig. 4:**
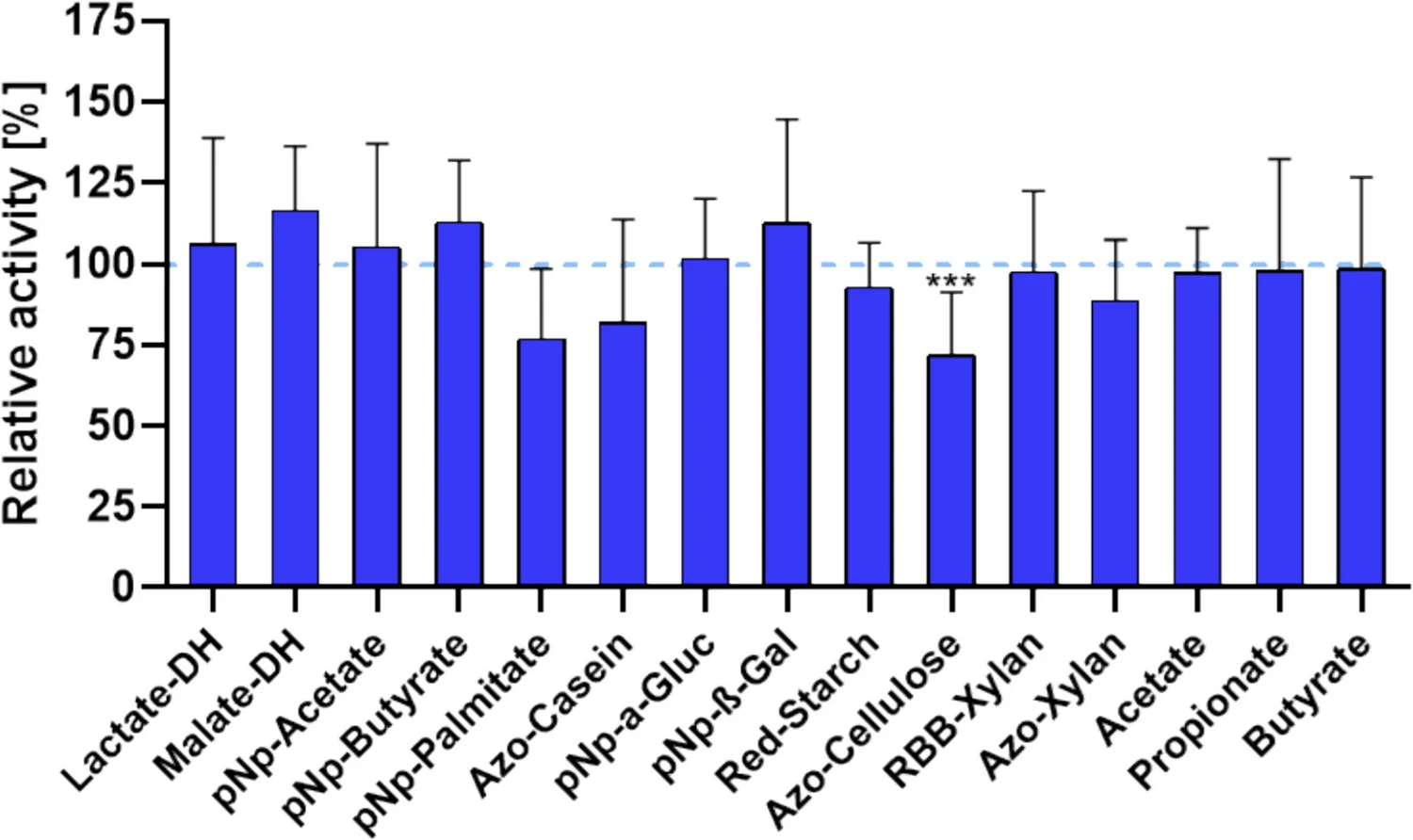
Relative enzymatic activities and SCFA production [%] of the bacterial colon model compared with original fecal samples. Specific enzymatic activities (nmol min⁻^1^ mg protein⁻^1^) were normalized to the corresponding fecal reference values, which were set to 100%. Bars represent the relative activity or concentration measured in the colon model after 24 h of incubation. DH = dehydrogenase, pNp = p-nitrophenyl, Azo = azo dye-coupled, RBB = Remazol Brilliant Blue-labelled, SCFA = short-chain fatty acids (acetate, propionate, butyrate). Statistical significance was determined using paired two-tailed *t*-tests (*n* = 8–10). Only Azo-cellulose showed a significant reduction (***p ≤ 0.001)

Based on the primary objective of restoring metabolic functionality while preserving microbial community structure, subsequent analyses focused on key enzymatic and fermentative pathways involved in core microbial metabolism. As the fiber-rich substrate formulation was most effective in maintaining the community composition of the bacterial colon model, this condition was selected to assess microbial enzymatic activity after 24 h. Enzymatic activities were compared to those of diluted fecal reference samples to determine the degree of functional similarity between the in vitro model and the native gut microbiota. The analyses encompassed a representative panel of metabolic enzymes, including intracellular oxidoreductases (lactate dehydrogenase, malate dehydrogenase), hydrolases such as esterases, lipases, and proteases, as well as carbohydrate-active enzymes (α-glucosidase, β-galactosidase, β-xylosidase, cellulase, and xylanase). In addition, the main microbial fermentation products, acetate, propionate, and butyrate were quantified to assess overall fermentative capacity and metabolic output (Fig. 4).

The activities of key intracellular redox enzymes, lactate- and malate-dehydrogenase, were maintained or slightly elevated relative to fecal reference values (lactate DH 106%; malate DH: 116%), indicating preserved intracellular metabolism. A similar pattern was observed for the esterases catalyzing the hydrolysis of acetate and butyrate esters, with pNp-acetate and pNp-butyrate activities slightly exceeding the fecal reference values (105% and 113%, respectively). In contrast, enzymes associated with lipid and protein degradation exhibited slightly reduced values. Lipolytic activity, measured by pNp-palmitate hydrolysis, was decreased to 77%, and proteolytic activity, assessed by azo-casein hydrolysis, showed activity levels at 82% of the fecal control. These reductions likely do not reflect a general metabolic impairment but rather substrate-induced regulatory shifts. The absence of complex dietary lipids and proteins in the incubation medium may have downregulated lipase and protease expression, thereby redirecting e microbial metabolism toward carbohydrate utilization. 

Among glycoside hydrolases, a particularly consistent recovery was evident for enzymes mediating glycosidic bond cleavage. α-Glucosidase and β-galactosidase activities even exceeded fecal reference levels (102% and 113%, respectively), highlighting an efficient re-establishment of saccharolytic functionality. Enzymes involved in the breakdown of structurally complex polysaccharides showed a largely preserved activity profile relative to fecal reference levels. The α-amylase activity, determined with red starch as the substrate, remained close to baseline at 93%, while xylanolytic activity showed only marginal reductions, reaching 98% and 89% of reference values with RBB- and Azo-xylan, respectively. In contrast, cellulase activity was significantly lower at 72% of the fecal control (*p* ≤ 0.001), indicating a partial loss of cellulose-degrading capacity. Similar to lipases and proteases, this reduction likely reflects the absence of corresponding structural polysaccharides in the incubation medium, resulting in downregulation of the respective hydrolases. The SCFA production, as an indicator of microbial fermentation, remained stable across all three major end products (acetate, propionate, and butyrate) with levels around 100% in comparison to fecal samples.

Taken together, these results demonstrate that the bacterial colon model supports robust bacterial metabolic activity closely reflecting that of the original fecal microbiota. Moreover, the data highlight the critical influence of substrate composition in shaping not only the taxonomic structure but also the functional capacity of the gut community under in vitro conditions. The overall enzymatic potential remained high, indicating that key metabolic functions were largely preserved. This suggests that the model also retains the broad enzymatic repertoire required for more complex metabolic transformations, such as xenobiotic modification.

#### Substrate supplementation is essential to preserve physiological enzyme activities in the in vitro colon model

**Fig. 5:**
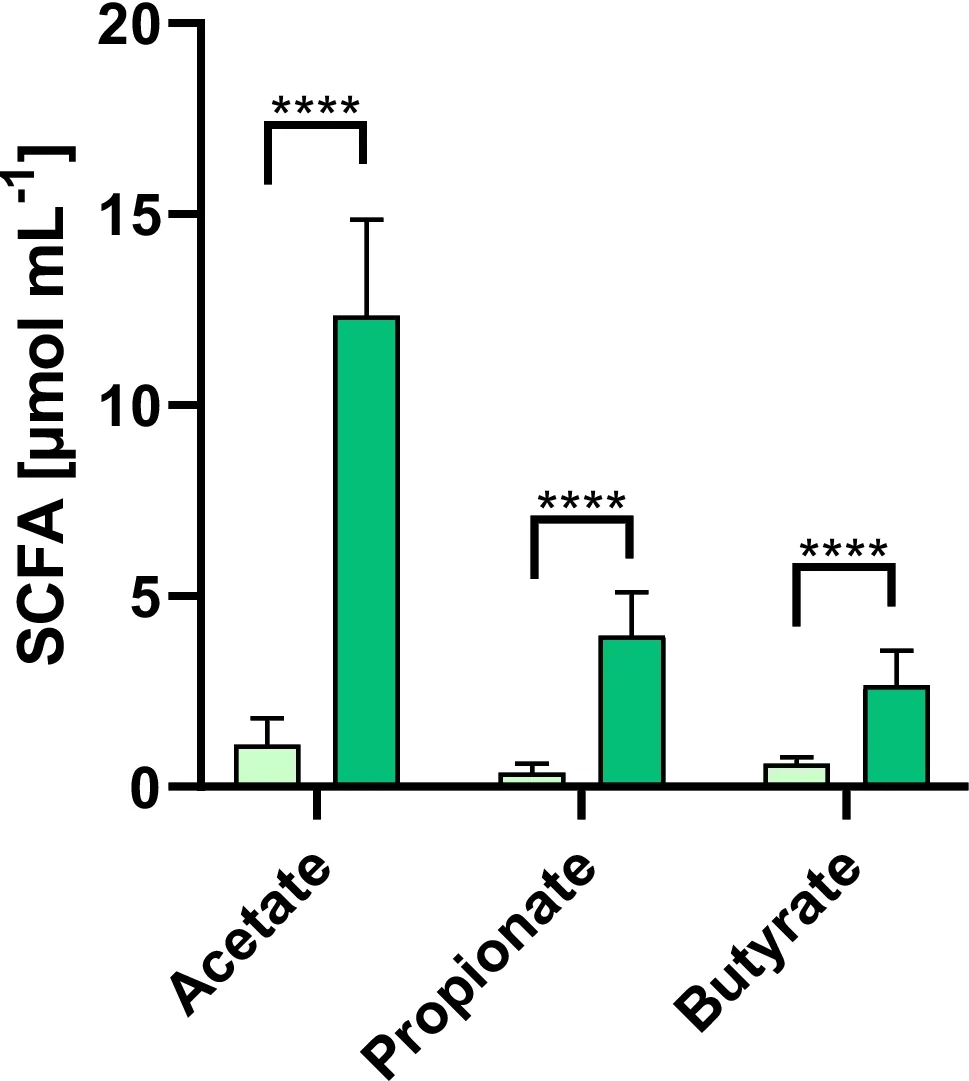
SCFA production in the bacterial colon model with and without substrate supplementation. Concentrations of acetate, propionate, and butyrate after 24 h of anaerobic incubation of the colon model without substrate (light green) or with the fiber-rich formulation (dark green). SCFAs were quantified by HPLC and expressed as µmol mL⁻^1^. Substrate addition markedly increased SCFA formation compared to the substrate-free control (****p ≤ 0.0001, paired *t*-test)

To assess the extent to which microbial functionality depends on substrate availability, SCFA concentrations were quantified in colon model samples following 24 h of incubation with and without supplementation of the fiber-rich substrate. The results clearly demonstrate that the presence of polysaccharides is critical for the production of the major fermentative end products (acetate, propionate, and butyrate). SCFA levels increased by approximately one order of magnitude compared to the substrate-free control, and all differences were highly significant (****p ≤ 0.0001). These findings indicate that physiologically relevant metabolic activity in the colon model can only be sustained in the presence of digestible substrates (Fig. 5).

**Fig. 6:**
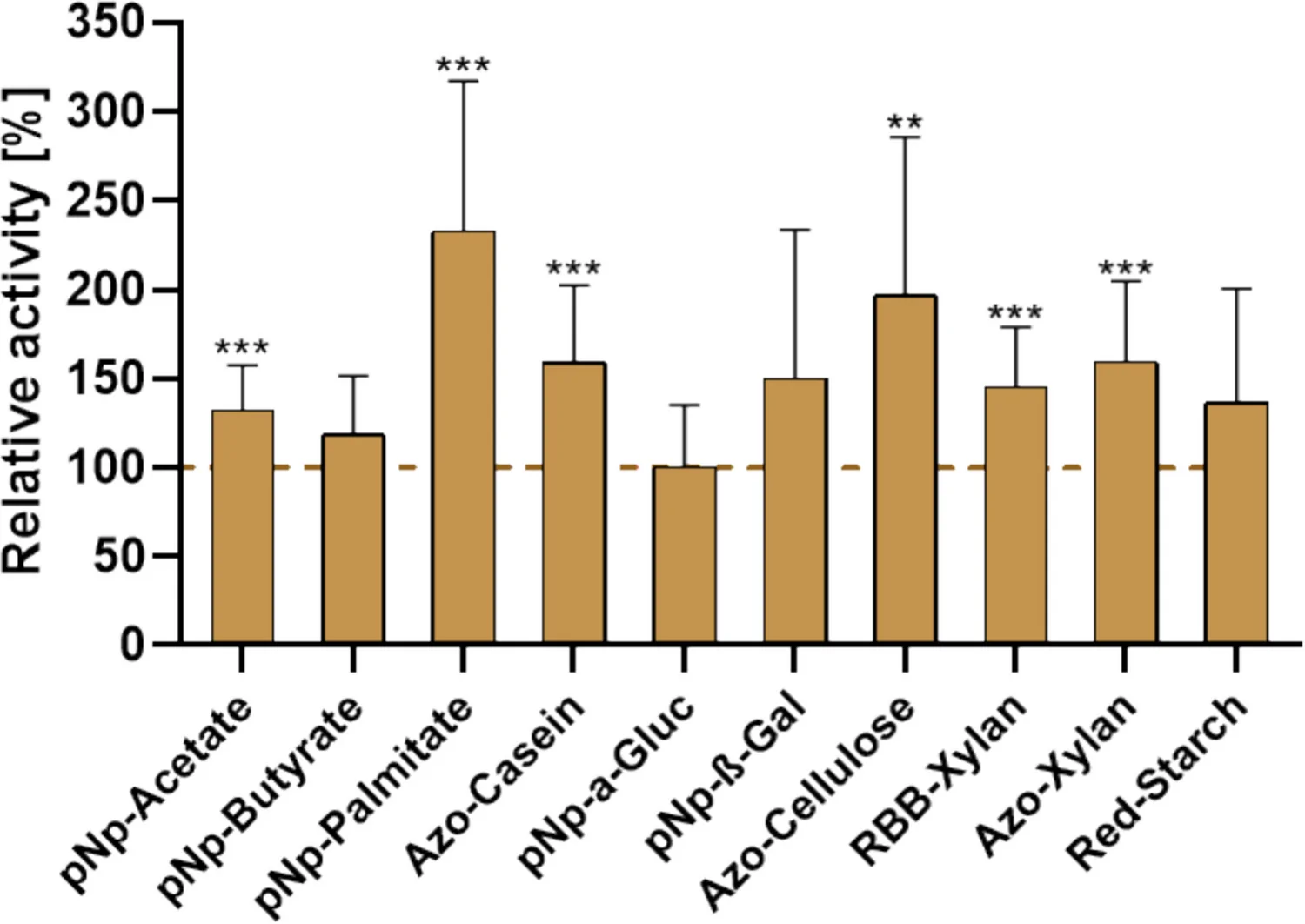
Enzymatic activity profile of the colon model at baseline (T0) and after incubation (T24) with the fiber-rich substrates. Activities are expressed as relative specific activities normalized to protein content (nmol min⁻^1^ mg protein⁻^1^), with T0 values set to 100%. Bars represent mean relative activities of key hydrolytic enzymes involved in microbial lipid, protein, and carbohydrate turnover. Significant increases were observed in several functional categories, including esterases, lipase, protease, glycoside hydrolases, and fibrolytic enzymes. Statistical evaluation was performed using paired *t*-tests (*n* = 8–10). Significance coding: ns (*p* > 0.05), *(*p* ≤ 0.05), **(*p* ≤ 0.01), ***(*p* ≤ 0.001), ****(*p* ≤ 0.0001)

Given that washing effectively removes extracellular enzymes, further experiments focused on the model’s capacity to regain functional activity in response to substrate availability. Representative hydrolytic enzyme activities were quantified at baseline (T0) and after 24 h of incubation with the fiber-rich substrate (T24) (Fig. 6). This targeted approach provides a direct evaluation of substrate-dependent metabolic adaptability, revealing the extent to which key enzymatic functions can be re-established in vitro.

After incubation, the colon model showed a significant reactivation of microbial enzymatic functions, indicating a shift from a metabolically suppressed to a physiologically active state. Compared with baseline (T0), mean relative hydrolytic activities increased across all enzyme classes, ranging from marginal changes (< 1%) to more than twofold baseline levels (> 100%). The most significant changes were observed in enzymes associated with polymer and lipid degradation. Lipase activity showed the strongest response, increasing more than twofold to 233% of baseline levels, while proteolytic activity also increased to 159%. Esterase activities showed significant upregulation, with pNp-acetate and pNp-butyrate hydrolysis reaching 132% and 119% of baseline values, respectively. Within the glycoside hydrolase group, β-galactosidase activity increased by approximately 50%, whereas α-glucosidase activity remained unchanged, consistent with the notion that some substrates are also accessible to intracellular enzymes, resulting in a lower apparent regeneration requirement. Enzymes involved in the breakdown of complex polysaccharides responded particularly strongly: cellulase activity nearly doubled to 197%, while xylanase activities increased by 45–59% with both xylan substrates tested. In contrast, α-amylase activity increased moderately to 136%. Taken together, these data demonstrate that the metabolic capacity of microbes in the colon model remained largely latent in the absence of environmental stimulation but could be reactivated by providing the appropriate substrate. This emphasizes the vital role of nutrition in restoring physiologically relevant microbial activity.

#### Functional validation of the bacterial colon model using microbial xenobiotic metabolism

**Fig. 7:**
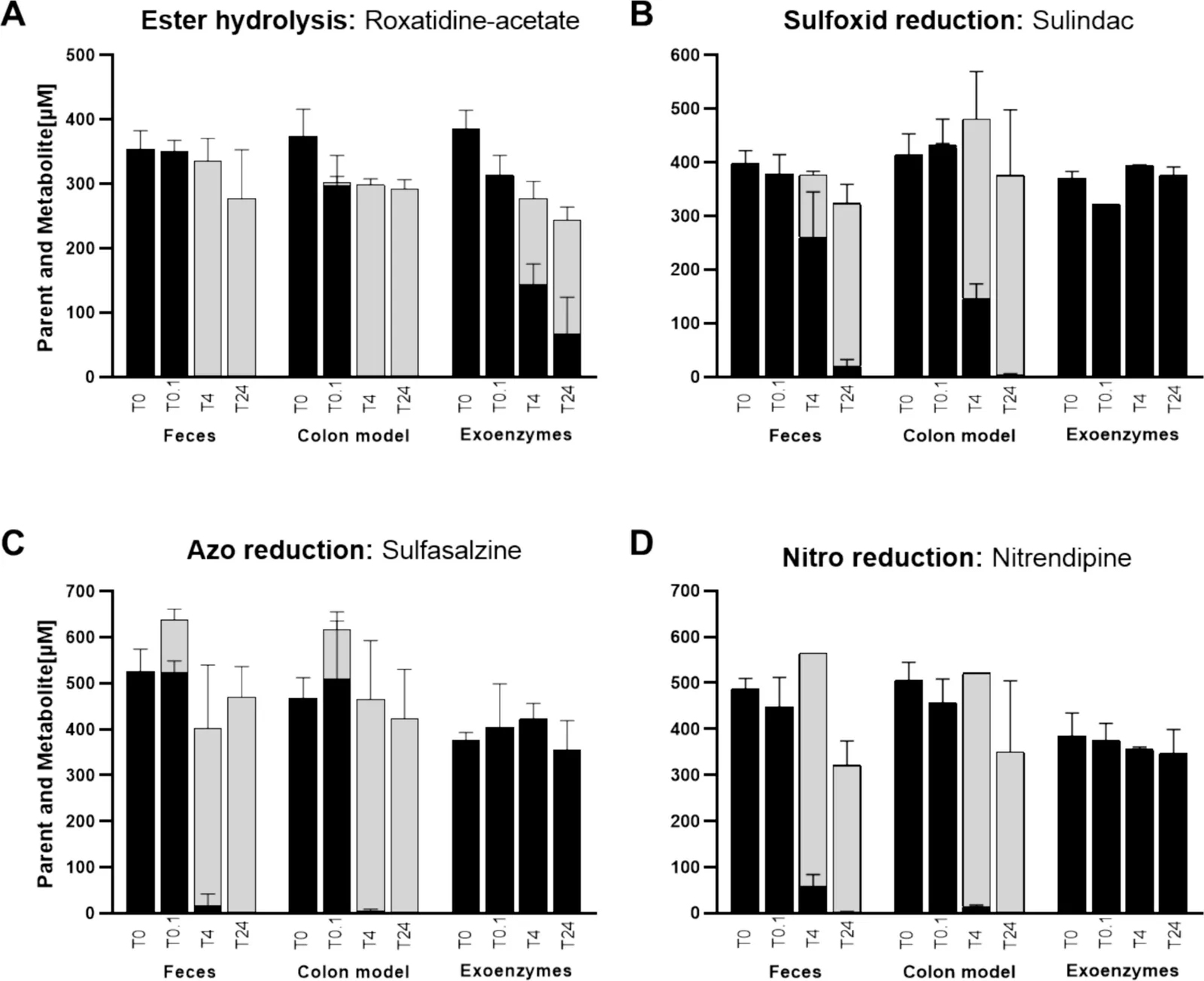
Microbial conversion of xenobiotic model compounds in buffer, fecal slurry, colon model, and the corresponding exoenzyme fraction. Representative substrates were selected to cover different microbial transformation reactions commonly observed in the human colon: roxatidine acetate for ester hydrolysis (**A**), sulindac for sulfoxide reduction (**B**), sulfasalazine for azoreduction (**C**), and nitrendipine for nitroreduction (**D**). Black bars indicate precursor compound concentrations, while grey bars stacked on top of the black bars represent the respective metabolite concentrations, quantified by HPLC. Quantification was performed using compound-specific calibrations where available, for nitrendipine, product levels were estimated using the precursor calibration (semi-quantitative). Data represent mean ± SD of three independent biological pools, each composed of three human donors

To evaluate the metabolic capabilities of the human colon model presented here, a panel of structurally diverse xenobiotics was selected based on microbial biotransformation reactions reported in the literature (Fig. 7, Supplementary Fig. S2). A total of six representative compounds covering four major reaction classes were included: ester hydrolysis, represented by roxatidine-acetate (Fig. 7A) (Zimmermann et al. 2019) and 4-acetoxyacetanilide (Supplementary Fig. S2) (Kamberi et al. 2004), sulfoxide reduction, using sulindac (Fig. 7B) (Lemmens et al. 2021), azoreduction, exemplified by sulfasalazine (Fig. 7C) (Lima et al. 2024), as well as nitroreduction using nitrendipine (Fig. 7D) (Vertzoni et al. 2018) and chloramphenicol (Supplementary Fig. S2) (Crofts et al. 2019). Together, these reference substrates served as model compounds covering major gut microbial transformation chemistries, enabling a matrix-to-matrix performance evaluation of the colon model. All compounds were incubated anaerobically for 24 h with pooled human fecal slurry, the bacterial colon model, and the sterile-filtered fecal exoenzyme fraction, derived from three independent donor pools (*n* = 3 each). For substrates with available standards, precursor compound depletion and the formation of the corresponding products were quantified, yielding combined precursor and product recoveries ranging from 78 to 100%. The apparent first-order kinetic parameters derived from precursor compound depletion are summarized in Supplementary Table S4. Roxatidine-acetate showed rapid depletion with a parallel increase in roxatidine, indicating efficient ester hydrolysis. Identity was supported by LC–MS signals at m/z 349.2111 (roxatidine acetate) and m/z 307.2015 (roxatidine). Apparent turnover was nearly identical in the fecal slurry and colon model, with half-lives of 0.60 and 0.61 h, whereas conversion in the exoenzyme fraction was markedly slower (4.33 h) (Fig. 7). Reductive substrates converted more slowly with only minor product formation detectable during the first 4 h and pronounced depletion by 24 h. Sulindac (m/z 357.0948) conversion was consistent with sulfoxide reduction, as precursor depletion occurred alongside accumulation of the corresponding sulfide (m/z 341.0992). Turnover was faster in the colon model than in fecal slurry (half-lives 3.47 h and 5.78 h), while the exoenzyme fraction showed no meaningful conversion, supporting an association with the intact microbial compartment. Sulfasalazine underwent rapid azo-bond cleavage with subsequent formation of sulfapyridine, accounting for most of the observed substrate loss. The precursor and primary product signals (m/z 399.0762 and 250.0630) showed close correlation, with a combined recovery of 80–100% across matrices. A low abundance signal at m/z 218.2117 suggested additional downstream metabolism but was below the quantification threshold . Conversion kinetics were similar in fecal slurry and the colon model (half-lives of 0.75 h and 0.58 h), while the exoenzyme fraction showed no detectable activity. Nitrendipine (m/z 359.1243) was nitro-reduced to the expected product signal at m/z 329.1519 (CAS No. 138135-48-5), with near-complete loss of the precursor compound. Product levels were estimated using the nitrendipine calibration and are therefore semi-quantitative. Turnover was faster in the colon model than in fecal slurry (half-livesof 0.75 h and 1.58 h), while the exoenzyme fraction showed no relevant conversion. Overall, degradation rates and kinetic profiles were comparable between fecal slurry and the colon model across the tested reaction classes, supporting preservation of key xenobiotic-transforming activities in the model. In contrast, the cell-free exoenzyme fraction showed measurable activity primarily toward ester substrates, consistent with the presence of extracellularly released hydrolytic enzymes, and provided complementary information on transformation processes that can proceed independently of intact cellular metabolism.

## Discussion

The gastrointestinal tract is a dynamic interface between host and environment, colonized by a complex microbial community whose metabolic potential exceeds that of the human organism (Thursby and Juge 2017). Models for studying gut microbiota dynamics and metabolism can be broadly categorized into three main classes: (i) static batch fermentations, (ii) continuous bioreactor systems, and (iii) microfluidic or organ-on-a-chip platforms. Each model type addresses different experimental needs and provides distinct levels of physiological representation. Dynamic multistage bioreactor systems, such as the Simulator of the Human Intestinal Microbial Ecosystem (SHIME) (Van De Wiele et al. 2015), the TNO Intestinal Model (TIM) (Minekus et al. 1999), the SIMulator of the GastroIntestinal tract (SIMGI) (Barroso et al. 2015), and the EnteroMix (Mäkeläinen et al. 2007), are designed to closely mimic the physicochemical gradients of the human gut. Although they offer high physiological relevance and spatial resolution, their complexity, infrastructure requirements, long stabilization periods, and limited scalability make them less suitable for high-throughput applications or routine screening studies. Microfluidic and organ-on-a-chip platforms, such as the Human-Microbial Crosstalk (HuMiX) model (Shah et al. 2016), the Human Oxygen-Bacteria Anaerobic (HoxBan) system (Sadaghian Sadabad et al. 2015), and the Host-Microbiota Interaction (HMI) model (Marzorati et al. 2014), allow controlled co-culture of gut epithelial cells and microbial communities. These systems provide valuable insights into host-microbe interactions, barrier integrity, and immunological signaling. However, simultaneously maintaining the aerobic requirements of the host epithelium and the strict anaerobic conditions required by gut microbes remains a major technical challenge. Furthermore, their complexity and low throughput limit their applicability to screening-based studies. Static batch fermentation systems constitute the most scalable and experimentally accessible model format. They are widely used for evaluating microbial transformations under anaerobic conditions, especially for studying specific metabolic activities or substrate conversion (Zimmermann et al. 2019; Shetty et al. 2022).

But a fundamental limitation shared by all current in vitro gut microbiota models is their reliance on either undefined fecal inocula (El Oufir et al. 2000; Marzorati et al. 2014) or simplified synthetic microbial communities composed of a limited number of strains (Goodman et al. 2009; van Leeuwen et al. 2023). Fecal samples represent a complex and undefined matrix containing not only a diverse bacterial population but also residual dietary material, host-derived cells, enzymes, and endogenous metabolites. This compositional complexity introduces substantial variability between preparations and complicates controlled experimental analyses, as test compounds may interact unpredictably with reactive components of the fecal matrix. The use of synthetic microbial consortia offers greater experimental standardization and reproducibility but tends to be limited in terms of taxonomic complexity (Goodman et al. 2009; Perez et al. 2021). Moreover, the use of fecal inocula, while taxonomically broad, often results in a loss of microbial diversity during prolonged incubation.

Although all existing gut microbiota models have their strengths and can be valuable tools in specific research contexts, their suitability must be critically evaluated in the context of the biological process under investigation. Especially in studies focusing on the microbial transformation of xenobiotics, it is essential that the models preserve both the full taxonomic and metabolic repertoire of the native gut microbiota while remaining compatible with high-throughput screening techniques. To overcome the limitations of existing in vitro approaches, we developed a novel bacterial colon model based on a purified suspension of native gut bacteria. Our system is built on a stable, high-density microbial community that closely reproduces the taxonomic diversity and metabolic functionality of the human colonic microbiota. Amplicon sequencing identified more than 400 bacterial species, highlighting the model’s ability to preserve a complex and representative microbial consortium over 24 h in a physiologically relevant dimension. Importantly, the preparation was free of fecal matrix components, including host cells, food residues, and reactive metabolites. A further advantage of this model is its modularity: microbiota preparations from different individuals can be combined to minimize stochastic effects and inter-individual outliers. However, the model is also fully compatible with single-donor preparations. This flexibility enables researchers to investigate donor-specific metabolic phenotypes if required. In addition, this platform supports microbial processing of dietary components, supplements, pharmaceuticals, and xenobiotics, with reduced confounding by substrate competition. This high level of analytical control makes the model particularly suitable for mechanistic studies at the interface of diet, xenobiotic metabolism, and microbial ecology. In parallel to other feces-derived models with a similar design, the bacterial colon model involves some trade-offs: as a static batch system, it does not reproduce key in vivo features such as spatial gradients (e.g., pH, transit, and absorption) or host-derived factors including mucus-associated niches, epithelial interactions, and immune modulation. Likewise, Nycodenz® separation and washing physically separate the microbiota from the native fecal matrix. While this may affect matrix-associated taxa and modify the local metabolite milieu, amplicon sequencing demonstrated a high overall similarity between native feces and the derived colon model, indicating that the major community structure was largely retained. Finally, the current study focuses on a 24 h screening window to enable high-throughput screening, to minimize community drift under batch conditions, and to reflect a physiologically relevant colonic transit time. Longer incubations are feasible but would likely introduce additional selective pressures and would require dedicated stabilization strategies. To ensure the physiological relevance of functional investigations, it is essential that microbial population densities approximate those found in the human colon. Native colonic communities reach approximately 8.8 × 10^10^ cells mL^−1^ (Sender et al. 2016), whereas many in vitro gut models operate at substantially lower bacterial concentrations (Minnebo et al. 2023), which can limit their capacity to reproduce in vivo-like metabolic activity. The bacterial colon model developed in this study addresses this limitation by employing an inoculum standardized to 1.43 × 10^10^ cells mL^−1^, with scalability up to 4.3 × 10^10^ cells mL^−1^. This corresponds to 50% of the in vivo bacterial density and therefore provides a realistic basis for analyzing microbial metabolism and community-driven biotransformations. At the same time, the system remains compatible with high-throughput formats, as assay volumes can be reduced to 100 µL, enabling efficient screening of large xenobiotic panels in multiwell plates. To ensure physiologically meaningful functionality, the model supports the regeneration of enzymatic activity, thereby maintaining the native community structure during incubation. Within 24 h, microbial functions including glycosidase, protease, and esterase activities were re-established to levels comparable to those measured in fecal reference samples. This allows short-term incubations (≤ 24 h) for the screening of bacterial transformations under stable compositional and biochemical conditions without the need for complex reactor systems or multi-day stabilization phases. In contrast, many established in vitro platforms require continuous cultivation and extended adaptation periods to achieve stable microbial community structures. The presented results also demonstrate that the metabolic activity of the gut microbiota can be functionally restored under defined in vitro conditions, provided that an appropriate substrate composition is selected. Comparative testing of different substrate formulations showed that fermentable carbohydrates are essential for restoring microbial metabolic functionality, yet the specific composition critically determines the extent of recovery. In particular, the fiber-rich formulation supported the most effective regeneration of enzymatic activities, preserved taxonomic stability, and enabled SCFA production at physiologically relevant levels. The recovered enzymatic activity in our bacterial colon model reflected that of fecal reference samples and fell within the physiologically range reported for native human feces. Literature values for key microbial enzymes typically span approximately 60–190 nmol min^−1^ mg protein ^−1^ (Flores et al. 2012; Śliżewska et al. 2023), placing the activities observed in our system well within the expected biological variability. Similarly, the concentrations of SCFAs produced in the bacterial colon model with 144.5 mmol kg feces^−1^ for acetate, 38.3 mmol kg feces^−1^ for propionate, and 30.7 mmol kg feces^−1^ for butyrate, were consistent with reported physiological ranges in human feces (acetate, 12.8–103.4 mmol kg⁻^1^; propionate, 4.5–27.8 mmol kg⁻^1^; butyrate, 4.0–53.0 mmol kg⁻^1^) (Høverstad et al. 1984). Notably, the proportional distribution of SCFAs in the model also mirrored the in vivo profile, which is typically characterized by relative ratios of approximately 60:20:20 for acetate, propionate, and butyrate, respectively (Cummings et al. 1987; Hijova and Chmelarova 2007; Binder 2010).

In contrast to many conventional systems, which must limit glycerol concentrations to 10–15% for cryopreservation (Bircher et al. 2020) to avoid introducing excess glycerol at inoculation, the presented model overcomes this limitation by incorporating a washing step that removes the cryoprotectant prior to experimental application. This allows the application of cryopreservation protocols using glycerol concentrations of 25% or higher, which are known to markedly improve cell viability and long-term stability (Tutrina and Zhurilov 2024). The removal of glycerol is particularly relevant because even small residual glycerol concentrations, as low as 1 mM, were found in preliminary experiments (data not shown) to substantially alter microbial fermentation profiles. Since glycerol serves as a metabolizable carbon source for fast-growing facultative anaerobes such as *E. coli* (Kopp et al. 2017), residual amounts can drive their selective overgrowth, thereby outcompeting slower-growing or more fastidious taxa. Such selective enrichment contributes to community imbalance and can fundamentally alter the system’s metabolic output (De Weirdt et al. 2010; Gao et al. 2021). The presented bacterial colon model offers considerable potential as a next-generation platform for translational microbiome research. It becomes feasible to conduct targeted comparisons between healthy and perturbed microbial communities, facilitating investigations into microbiome-associated disorders and their metabolic signatures. Currently, in-depth examinations of diet-microbiome interactions rely on in vivo studies, which are time-consuming, costly, and limited experimental (Wu et al. 2011; David et al. 2014). In contrast, the colon model permits studies on the effects of dietary components and supplements on microbial composition and metabolic output within a short timeframe that reflects the physiological transit time of the human colon. While the current work intentionally isolates microbiota-driven processes to provide a clean mechanistic readout, the system can be incorporated into host-microbiome frameworks. Coupling the bacterial colon model with gut-on-chip devices or in vivo mouse models would allow these platforms to benefit from its extensive taxonomic and functional diversity, thereby improving physiological fidelity in studies of host-microbial crosstalk. Beyond its applicability in mechanistic microbiome research, the presented model offers significant opportunities for the development of New Approach Methodologies (NAMs) aimed at reducing reliance on animal testing in toxicology. The system is also well suited to generate quantitative kinetic data for integration into physiologically based kinetic (PBK) models (Stevanoska et al. 2024). Although recent PBK frameworks have begun to incorporate microbial metabolism as a dedicated compartment, a major limitation remains the lack of standardized tools to characterize gut microbial transformation rates. By enabling controlled, high-resolution assessments of xenobiotic conversion, the bacterial colon model may help fill this gap and improve the predictive accuracy of in silico toxicokinetic simulations. Taken together, this system addresses a critical methodological gap by combining physiological complexity with experimental standardization, thereby providing a scalable and analytically robust platform for studying microbiota-mediated processes at the interface of diet, xenobiotic metabolism, and microbial ecology.

## Supplementary Information

Below is the link to the electronic supplementary material.

ESM 1 (PDF 1.25 MB)

## Data Availability

All sequencing data and processed datasets supporting the findings of this study are provided in the Supplementary Material. Raw datasets are available from the corresponding author upon reasonable request.

## Abbreviations

ACN: Acetonitrile
BS: Bile salts
BSA: Bovine serum albumin
CTAB: Cetyltrimethylammonium bromide
DNA: Deoxyribonucleic acid
EDTA: Ethylenediaminetetraacetic acid
EtOH: Ethanol
HMI: Host-microbiota interaction model
HoxBan: Human oxygen-bacteria anaerobic system
HPLC: High-performance liquid chromatography
HuMiX: Human-microbial crosstalk model
LC-MS: Liquid chromatography-mass spectrometry
LDH: Lactate dehydrogenase
LOD: Limit of detection
LOQ: Limit of quantification
MDH: Malate dehydrogenase
m/z: Mass-to-charge ratio
NADH: Nicotinamide adenine dinucleotide
NAMs: New approach methodologies
OD_600_: Optical density at 600 nm
PBK: Physiologically based kinetic
PBS: Phosphate-buffered saline
PCR: Polymerase chain reaction
pNp: P-nitrophenyl
RBB: Remazol Brilliant Blue
RI: Refractive index
SCFA: Short-chain fatty acids
SHIME®: Simulator of the Human Intestinal Microbial Ecosystem
SIMGI: Simulator of the gastrointestinal tract
T0: Baseline at 0 h
T24: Time point after 24 h incubation
TCA: Trichloroacetic acid
TIM: TNO intestinal model
UV: Ultraviolet

## References

[CR1] Abdelsalam NA, Ramadan AT, ElRakaiby MT, Aziz RK (2020) Toxico microbiomics: the human microbiome vs. pharmaceutical, dietary, and environmental xenobiotics. Front Pharmacol 11:390. 10.3389/fphar.2020.00390 32372951 10.3389/fphar.2020.00390 PMC7179069

[CR2] Barroso E, Cueva C, Peláez C, Martínez-Cuesta MC, Requena T (2015) Development of human colonic microbiota in the computer-controlled dynamic simulator of the gastrointestinal tract SIMGI. LWT Food Sci Technol 61:283–289. 10.1016/j.lwt.2014.12.014

[CR3] Binder HJ (2010) Role of colonic short-chain fatty acid transport in diarrhea. Annu Rev Physiol 72:297–313. 10.1146/annurev-physiol-021909-135817 20148677 10.1146/annurev-physiol-021909-135817

[CR4] Bircher L, Schwab C, Geirnaert A, Greppi A, Lacroix C (2020) Planktonic and sessile artificial colonic microbiota harbor distinct composition and reestablish differently upon frozen and freeze-dried long-term storage. mSystems 5:10.1128/msystems.00521-19. 10.1128/msystems.00521-19 10.1128/mSystems.00521-19 PMC6977070 31964766

[CR5] Boelsterli UA, Redinbo MR, Saitta KS (2013) Multiple NSAID-induced hits injure the small intestine: underlying mechanisms and novel strategies. Toxicol Sci 131:654–667. 10.1093/toxsci/kfs310 23091168 10.1093/toxsci/kfs310 PMC3551426

[CR6] Bradford MM (1976) A rapid and sensitive method for the quantitation of microgram quantities of protein utilizing the principle of protein-dye binding. Anal Biochem 72(1-2):248–254. 10.1016/0003-2697(76)90527-3 10.1016/0003-2697(76)90527-3 942051

[CR7] Callahan BJ, McMurdie PJ, Rosen MJ, Han AW, Johnson AJA, Holmes SP (2016) DADA2: high-resolution sample inference from Illumina amplicon data. Nat Methods 13:581–583. 10.1038/nmeth.3869 27214047 10.1038/nmeth.3869 PMC4927377

[CR8] Christ A, Lauterbach M, Latz E (2019) Western diet and the immune system: an inflammatory connection. Immunity 51:794–811. 10.1016/j.immuni.2019.09.020 31747581 10.1016/j.immuni.2019.09.020

[CR9] Clayton TA, Baker D, Lindon JC, Everett JR, Nicholson JK (2009) Pharmacometabonomic identification of a significant host-microbiome metabolic interaction affecting human drug metabolism. Proc Natl Acad Sci U S A 106:14728–14733. 10.1073/pnas.0904489106 19667173 10.1073/pnas.0904489106 PMC2731842

[CR10] Crofts TS, Sontha P, King AO, Wang B, Biddy BA, Zanolli N, Gaumnitz J, Dantas G (2019) Discovery and characterization of a nitroreductase capable of conferring bacterial resistance to chloramphenicol. Cell Chem Biol 26:559-570.e6. 10.1016/j.chembiol.2019.01.007 30799223 10.1016/j.chembiol.2019.01.007 PMC6474809

[CR11] Cummings JH, Pomare EW, Branch WJ, Naylor CP, Macfarlane GT (1987) Short chain fatty acids in human large intestine, portal, hepatic and venous blood. Gut 28:1221–1227. 10.1136/gut.28.10.1221 3678950 10.1136/gut.28.10.1221 PMC1433442

[CR12] David LA, Maurice CF, Carmody RN, Gootenberg DB, Button JE, Wolfe BE, Ling AV, Devlin AS, Varma Y, Fischbach MA, Biddinger SB, Dutton RJ, Turnbaugh PJ (2014) Diet rapidly and reproducibly alters the human gut microbiome. Nature 505:559–563. 10.1038/nature12820 24336217 10.1038/nature12820 PMC3957428

[CR13] De Weirdt R, Possemiers S, Vermeulen G, Moerdijk-Poortvliet TCW, Boschker HTS, Verstraete W, Van De Wiele T (2010) Human faecal microbiota display variable patterns of glycerol metabolism: glycerol metabolism in the human colon. FEMS Microbiol Ecol 74:601–611. 10.1111/j.1574-6941.2010.00974.x 20946352 10.1111/j.1574-6941.2010.00974.x

[CR14] Deloménie C, Fouix S, Longuemaux S, Brahimi N, Bizet C, Picard B, Denamur E, Dupret J-M (2001) Identification and functional characterization of arylamine *N* -acetyltransferases in eubacteria: evidence for highly selective acetylation of 5-aminosalicylic acid. J Bacteriol 183:3417–3427. 10.1128/JB.183.11.3417-3427.2001 11344150 10.1128/JB.183.11.3417-3427.2001 PMC99640

[CR15] El Oufir L, Barry J, Flourié B, Cherbut C, Cloarec D, Bornet F, Galmiche J (2000) Relationships between transit time in man and in vitro fermentation of dietary fiber by fecal bacteria. Eur J Clin Nutr 54:603–609. 10.1038/sj.ejcn.1600687 10951507 10.1038/sj.ejcn.1600687

[CR16] Flores R, Shi J, Gail MH, Ravel J, Goedert JJ (2012) Assessment of the human faecal microbiota: I. Measurement and reproducibility of selected enzymatic activities. Eur J Clin Invest 42:848–854. 10.1111/j.1365-2362.2012.02660.x 22409163 10.1111/j.1365-2362.2012.02660.x PMC3399928

[CR17] Gao Q, Li K, Zhong R, Long C, Liu L, Chen L, Zhang H (2021) Supplementing glycerol to inoculum induces changes in pH, SCFA profiles, and microbiota composition in in-vitro batch fermentation. Fermentation 8:18. 10.3390/fermentation8010018

[CR18] Gensollen T, Iyer SS, Kasper DL, Blumberg RS (2016) How colonization by microbiota in early life shapes the immune system. Science 352:539–544. 10.1126/science.aad9378 27126036 10.1126/science.aad9378 PMC5050524

[CR19] Goodman AL, McNulty NP, Zhao Y, Leip D, Mitra RD, Lozupone CA, Knight R, Gordon JI (2009) Identifying genetic determinants needed to establish a human gut symbiont in its habitat. Cell Host Microbe 6:279–289. 10.1016/j.chom.2009.08.003 19748469 10.1016/j.chom.2009.08.003 PMC2895552

[CR20] Hager N, Gindt ME, Hövels M, Dorne JCM, Deppenmeier U (2025) Distribution and activity of nitrate and nitrite reductases in the microbiota of the human intestinal tract. FEBS J. 10.1111/febs.70299 41189477 10.1111/febs.70299 PMC12998184

[CR21] Haiser HJ, Gootenberg DB, Chatman K, Sirasani G, Balskus EP, Turnbaugh PJ (2013) Predicting and manipulating cardiac drug inactivation by the human gut bacterium *Eggerthella lenta*. Science 341:295–298. 10.1126/science.1235872 23869020 10.1126/science.1235872 PMC3736355

[CR22] Hevia A, Delgado S, Margolles A, Sánchez B (2015) Application of density gradient for the isolation of the fecal microbial stool component and the potential use thereof. Sci Rep 5:16807. 10.1038/srep16807 26581409 10.1038/srep16807 PMC4652190

[CR23] Hijova E, Chmelarova A (2007) Short chain fatty acids and colonic health. Bratisl Lek Listy 108:354–358 18203540

[CR24] Høverstad T, Fausa O, Bjørneklett A, Bøhmer T (1984) Short-chain fatty acids in the normal human feces. Scand J Gastroenterol 19:375–381. 10.1080/00365521.1984.12005738 6740214

[CR25] Kamberi M, Riley CM, Ma X, Huang C-W (2004) A validated, sensitive HPLC method for the determination of trace impurities in acetaminophen drug substance. J Pharm Biomed Anal 34:123–128. 10.1016/j.japna.2003.08.015 14738926 10.1016/j.japna.2003.08.015

[CR26] Kopp J, Slouka C, Ulonska S, Kager J, Fricke J, Spadiut O, Herwig C (2017) Impact of glycerol as carbon source onto specific sugar and inducer uptake rates and inclusion body productivity in *E. coli* BL21(DE3). Bioengineering 5:1. 10.3390/bioengineering5010001 29267215 10.3390/bioengineering5010001 PMC5874867

[CR27] Koppel N, Maini Rekdal V, Balskus EP (2017) Chemical transformation of xenobiotics by the human gut microbiota. Science 356:eaag2770. 10.1126/science.aag2770 28642381 10.1126/science.aag2770 PMC5534341

[CR28] Lemmens G, Brouwers J, Snoeys J, Augustijns P, Vanuytsel T (2021) Insight into the colonic disposition of sulindac in humans. J Pharm Sci 110:259–267. 10.1016/j.xphs.2020.09.034 33002468 10.1016/j.xphs.2020.09.034

[CR29] Lima SF, Pires S, Rupert A, Oguntunmibi S, Jin W-B, Marderstein A, Funez-dePagnier G, Maldarelli G, Viladomiu M, Putzel G, Yang W, Tran N, Xiang G, Grier A, Guo C-J, Lukin D, Mandl LA, Scherl EJ, Longman RS (2024) The gut microbiome regulates the clinical efficacy of sulfasalazine therapy for IBD-associated spondyloarthritis. Cell Rep Med 5:101431. 10.1016/j.xcrm.2024.101431 38378002 10.1016/j.xcrm.2024.101431 PMC10982976

[CR30] Lindell AE, Zimmermann-Kogadeeva M, Patil KR (2022) Multimodal interactions of drugs, natural compounds and pollutants with the gut microbiota. Nat Rev Microbiol 20:431–443. 10.1038/s41579-022-00681-5 35102308 10.1038/s41579-022-00681-5 PMC7615390

[CR31] Macfarlane S, McBain AJ, Macfarlane GT (1997) Consequences of biofilm and sessile growth in the large intestine. Adv Dent Res 11:59–68. 10.1177/08959374970110011801 9524443 10.1177/08959374970110011801

[CR32] Mäkeläinen HS, Mäkivuokko HA, Salminen SJ, Rautonen NE, Ouwehand AC (2007) The effects of Polydextrose and Xylitol on microbial community and activity in a 4‐Stage colon simulator. J Food Sci. 10.1111/j.1750-3841.2007.00350.x 17995737 10.1111/j.1750-3841.2007.00350.x

[CR33] Marzorati M, Vanhoecke B, De Ryck T, Sadaghian Sadabad M, Pinheiro I, Possemiers S, Van Den Abbeele P, Derycke L, Bracke M, Pieters J, Hennebel T, Harmsen HJ, Verstraete W, Van De Wiele T (2014) The HMI™ module: a new tool to study the host-microbiota interaction in the human gastrointestinal tract in vitro. BMC Microbiol 14:133. 10.1186/1471-2180-14-133 24884540 10.1186/1471-2180-14-133 PMC4039060

[CR34] Minekus M, Smeets-Peeters M, Bernalier A, Marol-Bonnin S, Havenaar R, Marteau P, Alric M, Fonty G, Huis In’T Veld JHJ (1999) A computer-controlled system to simulate conditions of the large intestine with peristaltic mixing, water absorption and absorption of fermentation products. Appl Microbiol Biotechnol 53:108–114. 10.1007/s002530051622 10645630 10.1007/s002530051622

[CR35] Minnebo Y, De Paepe K, Raes J, Van de Wiele T (2023) Eating patterns contribute to shaping the gut microbiota in the mucosal simulator of the human intestinal microbial ecosystem. FEMS Microbiol Ecol 99:fiad149. 10.1093/femsec/fiad149 37974054 10.1093/femsec/fiad149

[CR36] Perez M, Ntemiri A, Tan H, Harris HMB, Roager HM, Ribière C, O’Toole PW (2021) A synthetic consortium of 100 gut commensals modulates the composition and function in a colon model of the microbiome of elderly subjects. Gut Microbes 13:1919464. 10.1080/19490976.2021.1919464 33993839 10.1080/19490976.2021.1919464 PMC8128205

[CR37] Qin J, Li R, Raes J, Arumugam M, Burgdorf KS, Manichanh C, Nielsen T, Pons N, Levenez F, Yamada T, Mende DR, Li J, Xu J, Li S, Li D, Cao J, Wang B, Liang H, Zheng H, Xie Y, Tap J, Lepage P, Bertalan M, Batto J-M, Hansen T, Le Paslier D, Linneberg A, Nielsen HB, Pelletier E, Renault P, Sicheritz-Ponten T, Turner K, Zhu H, Yu C, Li S, Jian M, Zhou Y, Li Y, Zhang X, Li S, Qin N, Yang H, Wang J, Brunak S, Doré J, Guarner F, Kristiansen K, Pedersen O, Parkhill J, Weissenbach J, Antolin M, Artiguenave F, Blottiere H, Borruel N, Bruls T, Casellas F, Chervaux C, Cultrone C, Delorme C, Denariaz G, Dervyn R, Forte M, Friss C, van de Guchte M, Guedon E, Haimet F, Jamet A, Juste C, Kaci G, Kleerebezem M, Knol J, Kristensen M, Layec S, Le Roux K, Leclerc M, Maguin E, Melo Minardi R, Oozeer R, Rescigno M, Sanchez N, Tims S, Torrejon T, Varela E, de Vos W, Winogradsky Y, Zoetendal E, Bork P, Ehrlich SD, Wang J, MetaHIT Consortium (2010) A human gut microbial gene catalogue established by metagenomic sequencing. Nature 464:59–65. 10.1038/nature08821 20203603 10.1038/nature08821 PMC3779803

[CR38] Sadaghian Sadabad M, Von Martels JZH, Khan MT, Blokzijl T, Paglia G, Dijkstra G, Harmsen HJM, Faber KN (2015) A simple coculture system shows mutualism between anaerobic faecalibacteria and epithelial Caco-2 cells. Sci Rep 5:17906. 10.1038/srep17906 26667159 10.1038/srep17906 PMC4678368

[CR39] Schmidt TSB, Raes J, Bork P (2018) The human gut microbiome: from association to modulation. Cell 172:1198–1215. 10.1016/j.cell.2018.02.044 29522742 10.1016/j.cell.2018.02.044

[CR40] Sender R, Fuchs S, Milo R (2016) Revised estimates for the number of human and bacteria cells in the body. PLoS Biol 14:e1002533. 10.1371/journal.pbio.1002533 27541692 10.1371/journal.pbio.1002533 PMC4991899

[CR41] Shah P, Fritz JV, Glaab E, Desai MS, Greenhalgh K, Frachet A, Niegowska M, Estes M, Jäger C, Seguin-Devaux C, Zenhausern F, Wilmes P (2016) A microfluidics-based in vitro model of the gastrointestinal human–microbe interface. Nat Commun 7:11535. 10.1038/ncomms11535 27168102 10.1038/ncomms11535 PMC4865890

[CR42] Shetty SA, Kuipers B, Atashgahi S, Aalvink S, Smidt H, De Vos WM (2022) Inter-species metabolic interactions in an in-vitro minimal human gut microbiome of core bacteria. Npj Biofilms Microbiomes 8:21. 10.1038/s41522-022-00275-2 35395818 10.1038/s41522-022-00275-2 PMC8993927

[CR43] Śliżewska K, Włodarczyk M, Sobczak M, Barczyńska R, Kapuśniak J, Socha P, Wierzbicka-Rucińska A, Kotowska A (2023) Comparison of the activity of fecal enzymes and concentration of SCFA in healthy and overweight children. Nutrients 15:987. 10.3390/nu15040987 36839343 10.3390/nu15040987 PMC9966664

[CR44] Smacchi E, Gobbetti M (2000) Bioactive peptides in dairy products: synthesis and interaction with proteolytic enzymes. Food Microbiol 17:129–141. 10.1006/fmic.1999.0302

[CR45] Soucek P (2011) Xenobiotics. In: Schwab M (ed) Encyclopedia of Cancer. Springer, Berlin Heidelberg, Berlin, Heidelberg, pp 3964–3967

[CR46] Statovci D, Aguilera M, MacSharry J, Melgar S (2017) The impact of Western diet and nutrients on the microbiota and immune response at mucosal interfaces. Front Immunol 8:838. 10.3389/fimmu.2017.00838 28804483 10.3389/fimmu.2017.00838 PMC5532387

[CR47] Stevanoska M, Folz J, Beekmann K, Aichinger G (2024) Physiologically based kinetic (PBK) modeling as a new approach methodology (NAM) for predicting systemic levels of gut microbial metabolites. Toxicol Lett 396:94–102. 10.1016/j.toxlet.2024.04.013 38685289 10.1016/j.toxlet.2024.04.013

[CR48] Takasuna K, Hagiwara T, Hirohashi M, Kato M, Nomura M, Nagai E, Yokoi T, Kamataki T (1996) Involvement of beta-glucuronidase in intestinal microflora in the intestinal toxicity of the antitumor camptothecin derivative irinotecan hydrochloride (CPT-11) in rats. Cancer Res 56:3752–3757 8706020

[CR49] Thursby E, Juge N (2017) Introduction to the human gut microbiota. Biochem J 474:1823–1836. 10.1042/BCJ20160510 28512250 10.1042/BCJ20160510 PMC5433529

[CR50] Tutrina A, Zhurilov P (2024) Efficacy assessment of different cryoprotectants for preserving the viability of Enterobacterales strains at − 20 °C. Sci Rep 14:20843. 10.1038/s41598-024-71529-6 39242800 10.1038/s41598-024-71529-6 PMC11379685

[CR51] Van De Wiele T, Van Den Abbeele P, Ossieur W, Possemiers S, Marzorati M (2015) The simulator of the human intestinal microbial ecosystem (SHIME®). In: Verhoeckx K, Cotter P, López-Expósito I, Kleiveland C, Lea T, Mackie A, Requena T, Swiatecka D, Wichers H (eds) The impact of food bioactives on health. Springer International Publishing, Cham, pp 305–317

[CR52] van Leeuwen PT, Brul S, Zhang J, Wortel MT (2023) Synthetic microbial communities (SynComs) of the human gut: design, assembly, and applications. FEMS Microbiol Rev 47:fuad012. 10.1093/femsre/fuad012 36931888 10.1093/femsre/fuad012 PMC10062696

[CR53] Vertzoni M, Kersten E, Van Der Mey D, Muenster U, Reppas C (2018) Evaluating the clinical importance of bacterial degradation of therapeutic agents in the lower intestine of adults using adult fecal material. Eur J Pharm Sci 125:142–150. 10.1016/j.ejps.2018.09.019 30273661 10.1016/j.ejps.2018.09.019

[CR54] Wu GD, Chen J, Hoffmann C, Bittinger K, Chen Y-Y, Keilbaugh SA, Bewtra M, Knights D, Walters WA, Knight R, Sinha R, Gilroy E, Gupta K, Baldassano R, Nessel L, Li H, Bushman FD, Lewis JD (2011) Linking long-term dietary patterns with gut microbial enterotypes. Science 334:105–108. 10.1126/science.1208344 21885731 10.1126/science.1208344 PMC3368382

[CR55] Yüksel E, Kort R, Voragen AGJ (2025) Structure and degradation dynamics of dietary pectin. Crit Rev FoodSci Nutr 65:6249–6268. 10.1080/10408398.2024.2437573 10.1080/10408398.2024.2437573 39681562

[CR56] Zimmermann M, Zimmermann-Kogadeeva M, Wegmann R, Goodman AL (2019) Mapping humanmicrobiome drug metabolism by gut bacteria and their genes. Nature 570:462–467. 10.1038/s41586-019-1291-3 10.1038/s41586-019-1291-3 PMC6597290 31158845

