# Supplementary material for: Development of a gut microbiota model for the analysis of bacterial modifications of xenobiotics
Source: Appl Microbiol Biotechnol. 2026 May 30;110:163. doi: 10.1007/s00253-026-13861-7 (PMC13222238; doi:10.1007/s00253-026-13861-7)
Supplement: Supplementary file 1 [file 253_2026_13861_MOESM1_ESM.pdf]

Supplementary Material

Development of a gut microbiota model for the analysis of bacterial modifications of xenobiotics

Natalie Hager[1], Jan-Lorenz Weyers[1], Laura Falk[2], Maike Passon[2], Marie Christine Simon[3], Waldemar Seel[3,4], Jean-Lou Christian Michel Dorne[5], Uwe Deppenmeier[1*]

1) Institute of Microbiology and Biotechnology, University of Bonn, Meckenheimer Allee 168, 53115 Bonn, Germany

2) Institute for Food Chemistry, University of Bonn, Friedrich-Hirzebruch-Allee 7, 53115 Bonn, Germany

3) Institute for Nutrition and Microbiota, University of Bonn, Katzenburgweg 7, 53115 Bonn, Germany

4) NGS Core Facility, Institute for Human Genetics, University Hospital Bonn, Venusberg-Campus 1, 53127 Bonn, Germany

5) Methodology and Scientific Support Unit, European Food Safety Authority (EFSA), Via Carlo Magno 1A, 43126 Parma, Italy

***Corresponding author:** Uwe Deppenmeier; Institute of Microbiology and Biotechnology, University of Bonn, Meckenheimer Allee 168, 53115 Bonn, Germany; E-mail: udeppen@uni-bonn.de; Tel. +49-228-735590; Fax +49-228-737576; http://www.ifmb.uni-bonn.de/forschung/math.-nat.-fakultaet/ag-prof.-deppenmeier

Supplementary methods for xenobiotic transformation assays

All three media used in this work were based on a modified SHIME® salt solution. This solution served as a defined basal medium that replicated the ionic composition and buffering conditions of the intestinal lumen. The fiber-rich medium was used to represent a polysaccharide-rich colonic environment, whereas the Western-style media were designed to mimic a luminal environment richer in starch, protein, and fatty acids, reflecting dietary patterns associated with a Western-style diet. Supplementation with bile salts was included to additionally account for bile exposure under high-fat dietary conditions. Together, these media were selected to cover distinct physiologically relevant nutritional environments for functional regeneration of the isolated colon microbiota.

Table S1: Composition of the modified SHIME®-based regeneration media.

	Fiber-rich medium	Western-style medium without BS	Western-style medium with BS
$NaHCO_3$	29.8 mM	29.8 mM	29.8 mM
NaCl	34.2 mM	34.2 mM	34.2 mM
NH_4Cl	18.7 mM	18.7 mM	18.7 mM
K_2HPO_4	49.5 mM	49.5 mM	49.5 mM
KH_2PO_4	49.8 mM	49.8 mM	49.8 mM
$CaCl_2$ x 2 H_2O	0.07 mM	0.07 mM	0.07 mM
$MgSO_4$ x 7 H_2O	0.03 mM	0.03 mM	0.03 mM
Mucin	1 g L^{-1}	1 g L^{-1}	1 g L^{-1}
Pectin	1 g L^{-1}	1 g L^{-1}	1 g L^{-1}
Xylan	1 g L^{-1}	1 g L^{-1}	1 g L^{-1}
Starch	1 g L^{-1}	1 g L^{-1}	1 g L^{-1}
Casein	-	0.5 g L^{-1}	0.5 g L^{-1}
Peptone	-	0.5 g L^{-1}	0.5 g L^{-1}
Palmitic acid	-	0.25 g L^{-1}	0.25 g L^{-1}
Stearic acid	-	0.25 g L^{-1}	0.25 g L^{-1}
Oleic acid	-	0.25 g L^{-1}	0.25 g L^{-1}
Linoleic acid	-	0.25 g L^{-1}	0.25 g L^{-1}
Acetate	-	10 mM	10 mM
Propionate	-	10 mM	10 mM
Bile salts	-	-	0.5 g L^{-1}

To evaluate microbial xenobiotic metabolism under defined conditions, incubations were conducted using model substrates representing major biotransformation classes: 4-acetoxyacetanilide (ester hydrolysis), sulindac (sulfoxide reduction), sulfasalazine (azo bond reduction), and nitrendipine (nitro group reduction). Reaction mixtures were prepared under anaerobic conditions (37°C, 24 h) as follows: (i) 210 µL SHIME® saline containing the fiber-rich substrate formulation with 1 g L^{-1} mucin, pectin, and xylan (substrate control), (ii) 150 µL pooled fecal suspension (three individual pools with n = 3 donors each, 1:10 diluted in SHIME® saline) + 60 µL fiber-rich substrate, (iii) 300 µL pooled exoenzyme fraction (three individual pools with n = 3 donors each, 1:10 in SHIME® saline), and (iv) 150 µL bacterial colon model + 60 µL fiber-rich substrate. All incubations contained 500 µM of the respective test compound. At defined time points, samples were mixed 1:1 (v/v) with ethanol, vortexed, and centrifuged

(18,000 × g, 3 min). The resulting supernatant was combined 1:1 (v/v) with acetonitrile (100 %) and centrifuged again.

The clarified supernatant was diluted with the mobile phase prior to injection. Non-polar and UV-absorbing compounds were analyzed on a Knauer Eurospher II C8 column (250 × 4 mm, 5 µm) with a C8 guard cartridge using an AZURA CT 2 system (Knauer, Berlin, Germany) equipped with a SpectraSystem SCM1000 degasser, P4000 pump, AS3000 autosampler, and UVD 2.1L detector. Paracetamol was analyzed on a Shodex Asahipak NH2P-50 3E column (Showa Denko, Japan) with a corresponding guard column and an RI-101 detector (Ercatech AG, Germany). Chromatographic parameters, including mobile-phase composition, column temperature, flow rate, and detection wavelength are summarized in Suppl. Tab. S1. Quantification of parent compounds and metabolites was performed by external calibration using analytical reference standards.

Table S2: Chromatographic conditions for HPLC-based detection of selected xenobiotics and their microbial transformation products. Analytes were separated on either C8 or amino columns under compound-specific conditions. Mobile phases consisted of acetonitrile (ACN) with acidic modifiers and were operated under isocratic flow. Detection wavelengths correspond to the absorbance maxima of each compound.

	Column	Mobile phase	Tempera-ture [°C]	Flow rate [ml min^{-1}]	UV detection [nm]
**Sulindac**	C8	40 % ACN + 0.1 % H_3PO_4	40	0.5	340
**Sulindac-sulfide**	C8	40 % ACN + 0.1 % H_3PO_4	40	0.5	340
**Sulfasalazin**	C8	40 % ACN + 0.1 % H_3PO_4	40	0.5	340
**Sulfapyridine**	C8	15 % ACN + 0.1 % HCOOH	40	0.5	265
**Roxatidine-acetate**	C8	50 % KH_2PO_4 (50 mM) + 30 % MeOH + 20 % ACN pH 7.0	40	0.5	276
**Roxatidine**	C8	50 % KH_2PO_4 (50 mM) + 30 % MeOH + 20 % ACN pH 7.0	40	0.5	276
**4-Acetoxyacetanilide**	C8	40 % ACN	40	0.5	254
**Chloramphenicol**	C8	40 % ACN	40	0.5	254
**Paracetamol**	Amino	65 % ACN	60	0.5	240
**Nitrendipine**	C8	60 % ACN	40	0.5	254

To confirm the identity of selected biotransformation products, representative samples were analyzed by liquid chromatography-mass spectrometry (LC-MS). Samples were mixed 1:1 (v/v) with ethanol and centrifuged (18,000 × g, 5 min). From the resulting supernatant, 50 µL were diluted in 450 µL H_2O: ACN (70:30, v/v). Prior to analysis, samples were filtered through 0.2 µm Chromafil RC-20/15 MS syringe filters. For chromatographic separation the Acquity

UPLC I-Class system (Waters, Milford, MA, USA), consisting of a binary pump, a sample manager cooled to 10°C and a column oven set at 40°C, was used. The system was equipped with an HSS T3 C18 column (2.1 × 100 mm; 1.8 µm) and a VanGuard HSS T3 C18 precolumn (5 × 2.1 mm, 1.7 µm) (Waters, Milford, MA, USA). Elution was performed with 0.1 % Formic acid in H_2O (A) and 0.1 % Formic acid in ACN (B) at a flow rate of 0.4 mL min^{-1}. The gradient elution was performed as follows: 0-1 min, 80 % B, 1-2.5 min, 50 % B, 2.5-3.7 min, 0 % B, 3.7-4.3 min, 0 % B, 4.3-5.0 min, 80 % B.

For mass spectrometric analysis the VION IMS QTOF MS was operated in MS full scan acquisition mode with an electrospray ionization (ESI) interface operating in both polarities (positive and negative). The capillary voltage was adjusted to 2.00 kV for both polarities. Nitrogen served as the desolvation, nebulizing, and collision gas. The source temperature was maintained at 120°C, while the desolvation gas temperature was set to 550°C with a flow rate of 700 L h^{-1}.

Scan range was 50-500 Da and calibration of the instrument was conducted on a weekly basis using the “Major Mix” (Waters, Milford, MA, USA). For automated mass correction, a leucine-enkephalin solution (100 pg μL^{-1}) was used to ensure consistent mass accuracy throughout the analyses. The data was acquired and processed with UNIFI v1.9.2.045 (Waters, Milford, MA, USA).

Supplementary comparison of microbial community composition in native feces and the derived colon model

To assess whether Nycodenz®-based purification altered the microbial community composition, native fecal samples before purification and the corresponding bacterial colon model obtained after density-gradient centrifugation were compared by 16S rRNA gene amplicon sequencing (Supplementary Fig. S1). Overall, no significant differences were observed between both samples. Alpha-diversity parameters were highly comparable between native feces and the colon model. Specifically, the number of observed features amounted to 374.8 ± 83.5 in native feces and 353.0 ± 63.7 in the colon model (H = 0.273, p = 0.602, q = 0.667). Likewise, Shannon diversity was similar in both groups (6.50 ± 0.24 and 6.37 ± 0.09, H = 2.455, p = 0.117, q = 0.353), as were Pielou’s evenness (0.763 ± 0.019 and 0.755 ± 0.018, H = 0.535, p = 0.465, q = 0.655) and Faith’s phylogenetic diversity (19.700 ± 3.673 and 19.188 ± 3.299, H = 0.098, p = 0.754, q = 0.782) in native feces and the colon model, respectively. Beta-diversity analyses based on Bray-Curtis, Jaccard, weighted UniFrac, and unweighted UniFrac distances likewise did not show a significant separation between native feces and the bacterial colon model. The mean pairwise distances between corresponding native fecal samples and the colon model fractions were 0.153 ± 0.032 for Bray-Curtis, 0.265 ± 0.055 for Jaccard, 0.053 ± 0.021 for weighted UniFrac, and 0.146 ± 0.014 for

unweighted UniFrac. Pairwise comparison of both groups resulted in pseudo-F = 0.158, p = 0.892, q = 0.892 for Bray-Curtis, pseudo-F = 0.167, p = 0.896, q = 0.896 for Jaccard, pseudo-F = 0.496, p = 0.762, q = 0.762 for weighted UniFrac, and pseudo-F = 0.136, p = 0.918, q = 0.918 for unweighted UniFrac. Thus, neither abundance-based nor phylogeny-based distance measures indicated a detectable global shift in community structure. This was further supported by genus-level composition profiles, which remained highly similar after Nycodenz® enrichment when considering genera with a relative abundance > 0.5 %. Across the paired native feces and colon model samples, the dominant genera were consistently retained, including *Blautia*, *Faecalibacterium*, and *Bacteroides*, together with other abundant taxa such as *Fusicatenibacter*, *Gemmiger*, *Anaerostipes*, *Dorea*, and *Lachnospira*. Although moderate changes in the relative abundance of individual genera were observed in some sample pairs, the overall taxonomic profiles remained highly similar, with pairwise correlations ranging from r = 0.96 to 0.99. Together, these findings indicate that the purification procedure did not induce a detectable major shift in the overall microbial community composition and that the colon model largely preserved the original fecal microbiota structure while reducing matrix-derived background.

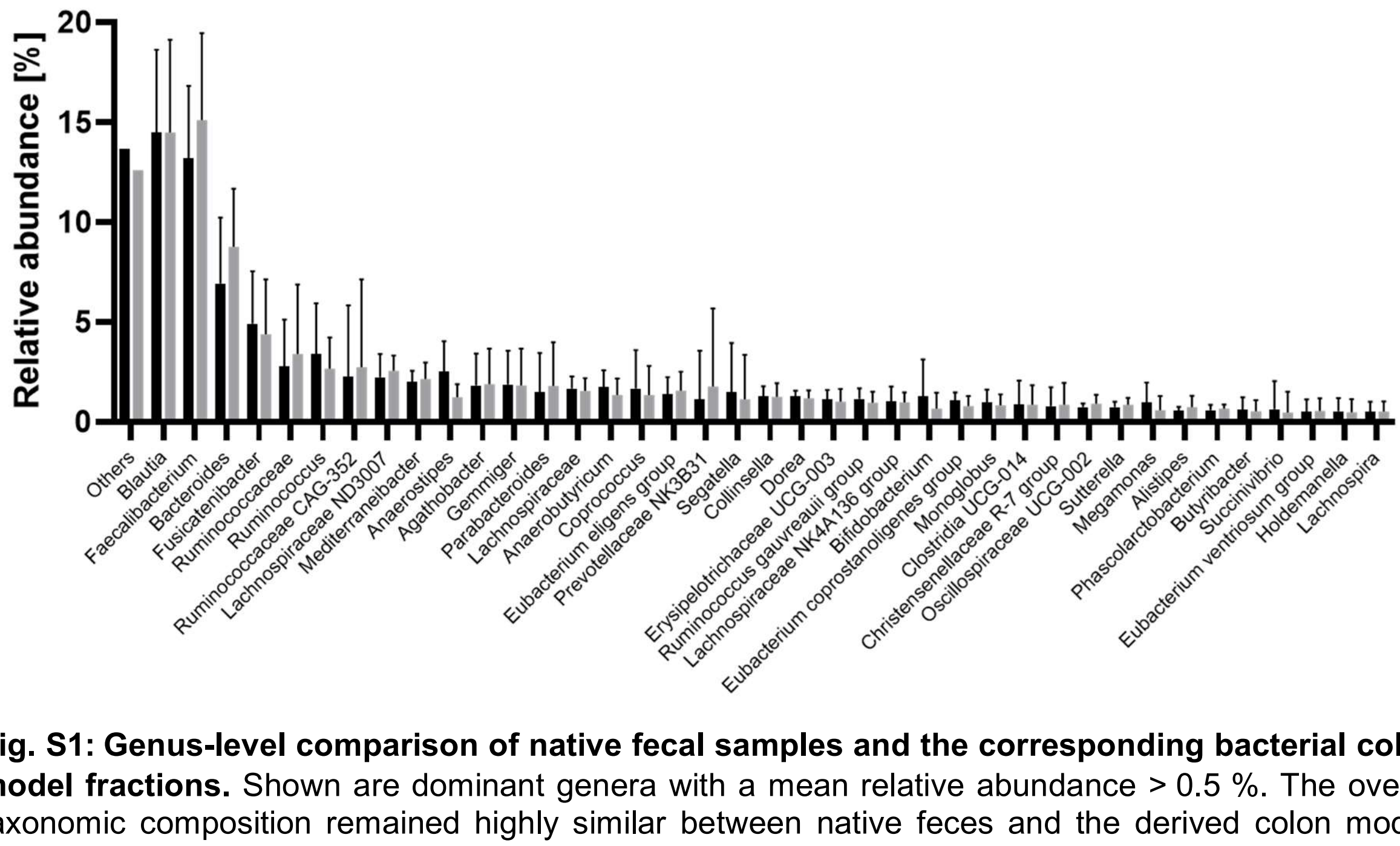

Fig. S1: Genus-level comparison of native fecal samples and the corresponding bacterial colon model fractions. Shown are dominant genera with a mean relative abundance > 0.5 %. The overall taxonomic composition remained highly similar between native feces and the derived colon model, indicating preservation of the original microbial community structure during Nycodenz®-based purification. In total, five paired sample pools were analyzed, with each native fecal pool (n=3) being compared to the corresponding bacterial colon model pool derived from the same starting material.

Supplementary dataset from 16S rRNA gene amplicon sequencing (Fig. 2)

Table S3: Genus-level hit counts from 16S rRNA gene amplicon sequencing of the bacterial colon model under different substrate conditions. Taxonomic assignments are based on high-quality reads after denoising and chimera removal (DADA2, Q ≥ 30). Only taxa with valid genus-level classification ("OK hits") are included. BS = Bile salts.

Hits	No Substrate T0 n=2 T24 n=3					Fiber-Rich T0 n=2 T24 n=3					Western + BS T0 n=2 T24 n=3					Western - BS T0 n=2 T24 n=3				
	**n=1**	**n=2**	**n=1**	**n=2**	**n=3**	**n=1**	**n=2**	**n=1**	**n=2**	**n=3**	**n=1**	**n=2**	**n=1**	**n=2**	**n=3**	**n=1**	**n=2**	**n=1**	**n=2**	**n=3**
Bacteroides	21970	9986	9948	9986	6810	10626	14124	9948	9986	6810	17233	14699	8766	7780	15898	13725	13016	26191	13484	18174
Bacteria	0	0	0	0	0	0	0	0	0	0	0	2	0	5	9	11	0	0	0	0
Enterobacteriaceae	0	0	19	0	8	0	0	19	0	8	0	0	620	372	915	0	0	68	15	37
Ruminococcaceae	207	418	449	418	311	97	108	449	418	311	138	99	123	104	238	67	100	389	395	480
Peptococcaceae	7	0	0	0	0	0	11	0	0	0	6	8	2	0	0	0	3	0	2	0
Lachnospiraceae_FCS020	322	352	381	352	241	135	179	381	352	241	234	197	141	87	229	156	158	132	153	166
DTU014	5	10	9	10	2	0	0	9	10	2	0	0	0	0	0	0	4	2	0	0
RF39	57	114	100	114	83	26	25	100	114	83	34	23	50	23	102	20	20	42	27	44
Faecalibacterium	22775	6462	6291	6462	4338	9849	13102	6291	6462	4338	14914	12728	3413	2215	5306	10992	11517	7522	6509	6690
Blautia	16152	13518	14182	13518	8952	6513	9132	14182	13518	8952	11558	8776	7369	5043	14912	6586	7785	7488	5419	6804
Dorea	1955	1941	2028	1941	1307	777	1031	2028	1941	1307	1258	1073	785	507	1506	653	990	965	700	948
[*Bacteroides*]_*pectinophilus*	30	39	44	39	11	20	15	44	39	11	17	13	9	0	10	13	11	16	6	16
Fournierella	37	123	135	123	94	0	0	135	123	94	36	33	0	9	45	37	28	92	150	161
Ruminococcaceae	110	128	119	128	104	51	66	119	128	104	66	62	35	0	66	52	67	71	57	57
Acetanaerobacterium	3	3	0	3	0	0	0	0	3	0	0	0	0	0	0	0	0	0	0	0
Lachnospira	1062	712	775	712	469	425	610	775	712	469	706	597	190	119	381	518	571	353	337	333
[*Eubacterium*]_*eligens*	1205	1184	1224	1184	818	495	676	1224	1184	818	803	643	713	524	1496	654	604	1032	717	901
Lachnospiraceae	2124	1791	1980	1791	1171	986	1155	1980	1791	1171	1377	1197	693	585	1591	918	1012	920	696	1015

Hits	No Substrate T0 n=2 T24 n=3					Fiber-Rich T0 n=2 T24 n=3					Western + BS T0 n=2 T24 n=3					Western - BS T0 n=2 T24 n=3				
	n=1	n=2	n=1	n=2	n=3	n=1	n=2	n=1	n=2	n=3	n=1	n=2	n=1	n=2	n=3	n=1	n=2	n=1	n=2	n=3
Lachnospiraceae_ND3007	4574	3618	3769	3618	2594	2046	2588	3769	3618	2594	3017	2740	1691	1177	3360	2532	2361	1987	1464	1476
Sutterella	1876	622	558	622	430	925	1185	558	622	430	1416	1315	1067	798	2229	1146	1152	1185	2085	1189
Ruminococcus	2115	1635	1420	1635	1197	988	1188	1420	1635	1197	1192	1015	324	271	609	896	1080	502	526	467
Lachnospiraceae	0	0	0	0	0	0	2	0	0	0	0	0	0	0	0	0	0	0	0	0
[*Eubacterium*]_*nodatum*	0	0	11	0	0	0	3	11	0	0	0	9	0	0	0	0	0	0	9	12
Anaerovoracaceae	175	168	150	168	151	43	67	150	168	151	98	76	44	27	88	72	65	212	114	160
Lachnospiraceae	526	649	712	649	396	212	324	712	649	396	319	302	197	179	538	190	217	296	375	404
Romboutsia	77	56	64	56	38	40	38	64	56	38	33	39	55	26	98	43	33	38	34	29
Intestinibacter	44	38	29	38	12	20	26	29	38	12	25	21	17	4	25	15	24	16	16	20
Terrisporobacter	17	13	16	13	8	0	1	16	13	8	9	0	15	11	21	0	0	0	8	11
Eubacterium	877	1325	1274	1325	838	387	555	1274	1325	838	638	594	556	407	1193	383	496	745	658	666
Lachnoclostridium	1630	2062	2085	2062	1428	654	932	2085	2062	1428	1110	936	2265	1602	4353	820	863	1928	1241	1926
Lachnospiraceae_UCG003	17	15	14	15	0	5	10	14	15	0	12	12	0	0	8	9	18	7	7	8
Agathobacter	8962	7007	7545	7007	4624	3756	4905	7545	7007	4624	6025	5107	3133	2188	6196	4300	4401	3784	2993	3666
Actinomyces	0	8	8	8	0	0	0	8	8	0	5	0	0	5	8	0	0	2	0	4
Citrobacter	0	8	35	8	11	0	0	35	8	11	0	0	62	41	137	0	0	20	0	0
Enterobacter	8	0	9	0	0	0	0	9	0	0	6	9	345	213	446	0	10	59	27	36
Escherichia-Shigella	48	141	129	141	104	34	41	129	141	104	51	36	3540	3374	5846	33	39	6139	1003	3099
[*Eubacterium*]_*hallii*_group	3690	4537	4786	4537	3074	1593	2076	4786	4537	3074	2325	2081	4344	2723	8367	1939	1912	4808	3579	4462
Klebsiella	0	18	12	18	8	0	0	12	18	8	0	0	1795	1008	2709	0	0	92	7	56
Bifidobacterium	3047	3242	3400	3242	2474	1563	1828	3400	3242	2474	1861	1575	3356	2464	6601	1710	1653	3909	2765	2933
Phascolarctobacterium	1029	817	900	817	631	549	697	900	817	631	1381	1174	2297	1715	3922	772	722	1745	982	1316

Hits	No Substrate T0 n=2 T24 n=3					Fiber-Rich T0 n=2 T24 n=3					Western + BS T0 n=2 T24 n=3					Western - BS T0 n=2 T24 n=3				
	n=1	n=2	n=1	n=2	n=3	n=1	n=2	n=1	n=2	n=3	n=1	n=2	n=1	n=2	n=3	n=1	n=2	n=1	n=2	n=3
Kocuria	0	0	4	0	0	0	0	4	0	0	0	0	0	0	0	0	0	0	0	0
Anaerovoracaceae	56	64	58	64	43	39	40	58	64	43	38	27	38	21	66	34	34	65	51	47
Clostridia	0	0	0	0	0	0	0	0	0	0	0	0	0	0	0	0	0	2	0	0
Lachnospiraceae_UCG010	97	123	157	123	80	41	55	157	123	80	52	57	0	0	0	43	49	350	285	332
GCA-900066755	0	10	0	10	0	0	0	0	10	0	0	0	0	0	11	0	0	6	0	5
Tyzzerella	52	38	39	38	26	29	34	39	38	26	30	25	18	11	26	23	31	88	77	81
UCG-009	4	0	6	0	0	0	0	6	0	0	0	0	0	0	0	0	0	0	0	0
Lachnospiraceae NK4A136	1267	1127	1308	1127	804	516	684	1308	1127	804	809	690	100	79	259	426	508	405	474	475
Lachnospiraceae UCG001	920	525	531	525	308	383	532	531	525	308	628	548	149	118	303	496	397	211	114	180
Megasphaera	515	486	473	486	350	288	391	473	486	350	423	381	1824	1280	3719	372	353	434	325	341
Lachnospiraceae_NK4B4	54	24	24	24	22	18	26	24	24	22	35	12	0	0	8	28	20	11	11	10
Butyricicoccus	401	346	336	346	209	176	248	336	346	209	279	235	155	103	274	207	194	203	198	199
Erysipelotrichaceae	1652	1482	1483	1482	1131	829	1110	1483	1482	1131	1135	975	767	713	1869	938	909	1041	726	852
Clostridium_sensu _1	27	12	5	12	8	13	8	5	12	8	7	14	16	11	26	4	11	11	4	21
Paludicola	0	0	0	0	0	0	0	0	0	0	0	0	0	0	0	0	0	0	3	0
Finegoldia	3	0	0	0	0	0	0	0	0	0	0	0	0	0	0	0	0	0	0	0
Coprococcus	1629	2190	2411	2190	1496	690	960	2411	2190	1496	1065	958	583	503	1549	752	811	3075	1998	2834
Flavonifractor	117	191	160	191	96	68	87	160	191	96	72	67	42	16	126	67	73	98	101	82
[*Eubacterium*]_*ventriosum*	284	211	206	211	113	100	159	206	211	113	172	175	96	63	165	134	120	128	82	104
GCA-900066575	58	36	42	36	34	20	34	42	36	34	32	38	11	0	18	37	42	21	15	24
Lactonifactor	6	12	12	12	11	0	7	12	12	11	0	0	0	0	11	0	0	0	0	0
Frisingicoccus	7	39	47	39	32	4	0	47	39	32	0	4	0	0	9	0	0	18	14	28

Hits	No Substrate T0 n=2 T24 n=3					Fiber-Rich T0 n=2 T24 n=3					Western + BS T0 n=2 T24 n=3					Western - BS T0 n=2 T24 n=3				
	n=1	n=2	n=1	n=2	n=3	n=1	n=2	n=1	n=2	n=3	n=1	n=2	n=1	n=2	n=3	n=1	n=2	n=1	n=2	n=3
Catenibacillus	0	4	0	4	0	0	0	0	4	0	0	0	0	0	5	0	0	0	0	0
Subdoligranulum	4288	4387	4442	4387	3024	1885	2544	4442	4387	3024	2673	2436	3320	2214	6639	2338	2308	4029	3204	3443
[*Eubacterium*]_*fissicatena*	13	5	7	5	0	11	8	7	5	0	0	2	0	0	0	5	4	0	0	0
CHKCI001	0	0	0	0	0	0	0	0	0	0	3	0	0	0	0	0	0	0	0	0
Roseburia	3314	1560	1590	1560	967	1276	1668	1590	1560	967	2204	1698	267	214	610	1559	1529	974	879	1013
[*Ruminococcus*]_*torques*	1791	1731	1825	1731	1112	827	1026	1825	1731	1112	1190	976	785	547	1576	775	882	657	502	792
[*Ruminococcus*]_*gauvreauii*	895	695	708	695	427	338	454	708	695	427	558	508	258	127	297	341	425	55	149	132
Marvinbryantia	115	75	81	75	44	23	52	81	75	44	72	45	69	43	147	57	62	67	54	99
CAG-56	329	284	310	284	155	118	167	310	284	155	194	166	30	14	70	134	154	49	76	59
Shuttleworthia	7	10	0	10	7	0	8	0	10	7	5	0	13	0	10	16	0	8	0	0
Oscillospirales	0	6	4	6	2	0	0	4	6	2	0	0	0	0	0	7	0	3	3	0
Fusicatenibacter	6840	4688	4979	4688	3134	2816	3559	4979	4688	3134	4772	3958	1218	932	2190	3023	3364	1664	1238	1759
Fenollaria	0	0	0	0	2	0	0	0	0	2	0	0	0	0	0	0	0	0	0	0
[*Ruminococcus*]_*gnavus*	178	96	96	96	78	69	81	96	96	78	98	89	11	14	18	50	71	27	40	41
Christensenellaceae_R-7	948	1270	1289	1270	882	478	563	1289	1270	882	757	612	650	613	1523	461	478	1058	659	835
Candidatus_Soleaferrea	12	25	18	25	11	6	4	18	25	11	3	9	0	0	14	5	0	5	7	19
Anaerostipes	1711	1795	2028	1795	1227	775	958	2028	1795	1227	1177	1048	1642	1041	3319	903	866	1164	874	969
Eisenbergiella	5	8	11	8	0	0	6	11	8	0	0	0	10	20	60	0	0	28	13	23
Lachnospiraceae UCG008	0	0	6	0	0	0	0	6	0	0	0	0	0	0	0	0	0	0	0	0
Eubacterium xylanophilum	416	45	42	45	34	165	249	42	45	34	277	268	9	7	14	215	179	25	18	17
[*Eubacterium*]_*ruminantium*	374	447	488	447	300	149	231	488	447	300	190	169	45	45	94	128	172	241	235	226
Moryella	23	26	41	26	22	13	27	41	26	22	17	12	10	9	33	15	13	16	20	35

Hits	No Substrate T0 n=2 T24 n=3					Fiber-Rich T0 n=2 T24 n=3					Western + BS T0 n=2 T24 n=3					Western - BS T0 n=2 T24 n=3				
	n=1	n=2	n=1	n=2	n=3	n=1	n=2	n=1	n=2	n=3	n=1	n=2	n=1	n=2	n=3	n=1	n=2	n=1	n=2	n=3
UBA1819	28	29	18	29	20	11	10	18	29	20	22	14	0	6	39	12	17	26	21	14
Lachnospiraceae_NC2004	0	0	0	0	0	0	0	0	0	0	0	0	2	0	0	0	0	2	0	0
Enterorhabdus	0	3	11	3	5	0	0	11	3	5	7	0	6	5	12	6	4	0	8	5
Defluviitaleaceae UCG011	25	37	61	37	25	9	18	61	37	25	9	5	9	0	11	13	13	16	22	15
Oscillibacter	211	212	209	212	171	87	118	209	212	171	176	107	138	94	257	113	102	157	118	126
*Clostridia*_UCG-014	423	582	569	582	375	207	239	569	582	375	313	248	218	157	504	244	178	335	233	259
Desulfovibrionaceae	2	3	4	3	0	3	3	4	3	0	4	0	0	0	3	4	0	6	0	2
Desulfovibrio	8	12	7	12	6	4	8	7	12	6	5	10	20	13	18	7	4	7	16	9
Bilophila	207	227	231	227	169	128	134	231	227	169	163	121	204	165	397	127	104	239	158	167
Anaerofustis	5	3	0	3	0	2	4	0	3	0	0	3	0	0	0	8	2	0	3	7
UCG-008	26	15	21	15	7	18	12	21	15	7	8	6	20	6	20	12	6	18	10	9
Monoglobus	516	405	388	405	282	256	293	388	405	282	414	362	115	77	216	301	327	203	244	193
Haemophilus	16	6	11	6	7	8	10	11	6	7	15	13	0	0	0	4	9	4	0	4
Desulfovibrionaceae	3	10	4	10	3	0	3	4	10	3	3	0	5	4	5	6	0	0	0	3
Fusobacterium	332	79	75	79	45	173	282	75	79	45	257	228	1875	1431	3667	244	186	700	159	472
Marmoricola	0	0	0	0	0	0	0	0	0	0	0	0	0	0	0	0	0	0	0	0
Novosphingobium	0	4	0	4	0	0	0	0	4	0	0	0	0	0	0	0	0	0	0	0
Sphingomonadaceae	0	0	0	0	0	0	0	0	0	0	0	0	0	0	9	0	0	0	0	0
Pseudomonas	4	0	0	0	0	0	0	0	0	0	0	0	0	0	0	0	0	0	0	0
Hungatella	0	26	47	26	27	0	0	47	26	27	0	0	101	78	252	0	0	54	27	55
Roseomonas	0	0	0	0	0	0	0	0	0	0	0	0	0	0	3	0	0	0	0	0
Methylobacterium	0	0	0	0	0	0	0	0	0	0	0	0	0	0	5	0	0	0	0	0

Hits	No Substrate T0 n=2 T24 n=3					Fiber-Rich T0 n=2 T24 n=3					Western + BS T0 n=2 T24 n=3					Western - BS T0 n=2 T24 n=3				
	n=1	n=2	n=1	n=2	n=3	n=1	n=2	n=1	n=2	n=3	n=1	n=2	n=1	n=2	n=3	n=1	n=2	n=1	n=2	n=3
Enhydrobacter	2	0	4	0	0	0	0	4	0	0	0	0	0	0	0	0	0	0	0	0
Johnsonella	0	0	2	0	0	0	0	2	0	0	0	0	0	0	0	0	0	0	0	0
Clostridia_vadin BB60	34	72	60	72	48	13	17	60	72	48	16	6	0	0	0	10	13	32	54	38
Acinetobacter	0	0	0	0	0	0	0	0	0	0	0	0	0	0	0	0	0	0	2	0
UCG-010	37	78	61	78	36	13	21	61	78	36	34	27	6	4	25	22	20	26	32	19
Oscillospira	18	16	18	16	8	5	24	18	16	8	19	18	8	0	12	11	8	10	10	15
Oscillospiraceae	39	37	27	37	27	15	15	27	37	27	23	36	28	29	75	24	16	102	64	69
Rhodospirillales	31	9	14	9	6	14	21	14	9	6	20	23	3	0	0	20	15	7	6	0
Oscillospiraceae	252	230	236	230	146	110	137	236	230	146	154	149	85	42	160	130	109	164	150	112
Intestinimonas	27	46	64	46	42	6	22	64	46	42	23	16	6	15	11	10	6	44	20	28
NK4A214_group	433	638	598	638	388	222	294	598	638	388	401	329	170	144	368	298	311	603	365	461
Christensenellaceae	14	22	22	22	9	6	13	22	22	9	10	0	0	0	11	5	15	10	4	14
UCG-002	1958	3107	3019	3107	2175	916	1116	3019	3107	2175	1127	972	993	809	2119	959	1066	2566	1867	2302
UCG-003	708	794	828	794	557	381	391	828	794	557	525	410	432	345	671	322	421	614	357	439
Eubacterium oxidoreducen	0	0	0	0	0	0	0	0	0	0	0	0	0	0	0	2	0	0	0	0
Colidextribacter	340	285	246	285	174	143	212	246	285	174	193	153	73	39	146	161	189	159	160	137
Megamonas	1553	1672	1620	1672	1217	811	1045	1620	1672	1217	1069	899	1588	1094	2862	1033	845	1445	1016	1140
Pseudoflavonifractor	8	0	15	0	9	0	6	15	0	9	0	0	7	6	0	0	0	0	5	7
UCG-005	433	627	644	627	422	214	250	644	627	422	288	217	168	115	364	229	229	277	310	278
Christensenella	4	0	0	0	0	0	0	0	0	0	0	0	0	0	0	0	0	3	0	0
Papillibacter	6	0	0	0	0	0	0	0	0	0	0	0	0	0	5	0	0	0	0	0
Akkermansia	197	244	170	244	172	91	137	170	244	172	156	93	525	488	861	123	106	780	264	473

Hits	No Substrate T0 n=2 T24 n=3					Fiber-Rich T0 n=2 T24 n=3					Western + BS T0 n=2 T24 n=3					Western - BS T0 n=2 T24 n=3				
	n=1	n=2	n=1	n=2	n=3	n=1	n=2	n=1	n=2	n=3	n=1	n=2	n=1	n=2	n=3	n=1	n=2	n=1	n=2	n=3
Cloacibacillus	0	0	0	0	0	0	0	0	0	0	0	0	0	2	0	0	0	0	0	0
Lachnospiraceae UCG004	183	163	200	163	120	57	93	200	163	120	104	104	130	103	219	106	90	112	76	136
Prevotella	719	118	122	118	34	364	509	122	118	34	522	444	23	39	123	539	497	134	26	63
Muribaculaceae	106	31	38	31	32	58	61	38	31	32	85	74	0	5	3	59	47	9	16	9
Odoribacter	199	137	148	137	135	88	118	148	137	135	144	138	72	58	161	149	121	192	113	105
Alistipes	1400	5148	4893	5148	3881	727	884	4893	5148	3881	1058	917	5613	4296	11486	968	863	3252	1604	2229
Barnesiella	307	394	381	394	258	157	192	381	394	258	197	196	168	140	336	201	180	480	321	366
Parabacteroides	1454	1095	1170	1095	743	698	889	1170	1095	743	1176	980	3237	2696	6627	955	913	3107	1205	1877
Coprobacter	31	9	0	9	5	25	35	0	9	5	25	29	4	0	0	36	26	8	0	0
Holdemania	43	36	29	36	24	17	14	29	36	24	19	18	12	7	27	14	15	36	22	11
Butyricimonas	66	106	114	106	68	32	24	114	106	68	59	38	128	80	205	41	32	156	68	88
Barnesiellaceae	5	0	0	0	0	0	0	0	0	0	0	0	0	2	0	0	0	3	0	0
Sanguibacteroides	4	12	13	12	6	2	4	13	12	6	0	0	17	17	19	3	0	13	2	8
Rikenella	0	3	0	3	0	0	0	0	3	0	0	0	0	0	0	0	0	0	0	0
Paraprevotella	263	47	35	47	42	122	174	35	47	42	184	139	12	0	27	156	164	29	61	20
Puniceicoccaceae	14	17	36	17	20	9	9	36	17	20	8	9	37	23	69	9	6	56	24	30
Rhodobacteraceae	0	3	0	3	0	0	0	0	3	0	0	0	0	0	0	0	0	0	0	0
Paracoccus	0	0	0	0	0	0	0	0	0	0	0	0	0	0	0	0	0	2	0	0
Lactobacillus	24	30	34	30	39	24	14	34	30	39	29	27	36	19	51	21	21	29	19	23
Allisonella	5	0	0	0	0	5	2	0	0	0	0	5	25	10	43	4	5	7	0	0
Acidaminococcus	17	32	30	32	15	12	16	30	32	15	28	30	0	0	10	24	17	12	10	0
Veillonella	23	13	38	13	24	22	17	38	13	24	28	16	55	42	146	19	12	86	27	54

Hits	No Substrate T0 n=2 T24 n=3					Fiber-Rich T0 n=2 T24 n=3					Western + BS T0 n=2 T24 n=3					Western - BS T0 n=2 T24 n=3				
	n=1	n=2	n=1	n=2	n=3	n=1	n=2	n=1	n=2	n=3	n=1	n=2	n=1	n=2	n=3	n=1	n=2	n=1	n=2	n=3
Granulicatella	0	0	0	0	2	0	0	0	0	2	0	0	0	0	2	0	0	0	0	0
Dialister	241	204	227	204	123	133	148	227	204	123	199	188	195	141	342	144	137	165	158	160
Peptococcus	5	7	11	7	4	3	0	11	7	4	4	7	0	0	5	0	5	5	0	9
Lactococcus	5	3	3	3	0	0	0	3	3	0	0	7	3	0	0	0	5	4	3	5
Streptococcus	76	50	56	50	45	40	38	56	50	45	46	55	76	51	113	41	47	57	33	40
Turicibacter	35	38	27	38	22	16	29	27	38	22	35	29	53	25	81	27	30	39	24	25
Paenibacillaceae	3	0	0	0	0	0	0	0	0	0	0	0	0	0	0	0	0	0	0	0
[*Eubacterium*]_*brachy*	5	4	9	4	5	3	0	9	4	5	3	0	2	0	3	4	4	4	0	0
Olsenella	0	8	0	8	0	0	0	0	8	0	0	0	0	0	0	0	0	3	0	0
Collinsella	2169	1690	1684	1690	1229	1089	1339	1684	1690	1229	1451	1282	805	596	1706	1251	1191	1128	845	875
Enorma	4	5	0	5	0	0	0	0	5	0	0	0	5	6	8	0	0	0	0	0
Victivallis	0	0	0	0	0	0	0	0	0	0	0	0	0	0	0	2	4	0	0	0
Mastigocladopsis PC10914	0	0	0	0	0	0	0	0	0	0	0	0	0	0	0	0	0	0	0	0
Chloroplast	3	16	0	16	0	0	0	0	16	0	0	0	0	0	0	0	0	0	0	2
Anaeroplasma	13	10	11	10	3	3	4	11	10	3	11	10	0	0	0	12	3	0	0	0
Erysipelatoclostridium	63	42	48	42	20	10	26	48	42	20	22	16	26	11	47	28	19	30	16	16
Izemoplasmatales	0	22	13	22	14	0	3	13	22	14	4	4	0	0	0	3	7	0	0	4
Erysipelatoclostridiaceae	0	6	0	6	6	0	4	0	6	6	5	0	0	0	0	0	8	0	0	0
Dielma	0	0	0	0	0	0	0	0	0	0	0	0	0	0	0	0	0	0	0	0
[*Clostridium*]_*innocuum*	0	5	5	5	0	1	0	5	5	0	5	0	3	0	7	4	6	6	10	3
Merdibacter	4	0	4	0	8	0	5	4	0	8	8	0	0	3	6	5	3	6	0	0
Holdemanella	732	544	561	544	380	364	442	561	544	380	611	534	208	139	368	465	413	324	223	219

Hits	No Substrate T0 n=2 T24 n=3					Fiber-Rich T0 n=2 T24 n=3					Western + BS T0 n=2 T24 n=3					Western - BS T0 n=2 T24 n=3				
	**n=1**	**n=2**	**n=1**	**n=2**	**n=3**	**n=1**	**n=2**	**n=1**	**n=2**	**n=3**	**n=1**	**n=2**	**n=1**	**n=2**	**n=3**	**n=1**	**n=2**	**n=1**	**n=2**	**n=3**
Erysipelotrichaceae	0	0	0	0	0	0	0	0	0	0	0	0	0	0	0	5	0	0	0	0
Faecalitalea	33	38	34	38	18	7	19	34	38	18	24	16	21	15	34	14	15	24	14	20
Anaerococcus	3	0	0	0	0	0	0	0	0	0	0	0	0	0	0	0	0	0	0	0
Coriobacteriales	12	9	0	9	0	5	3	0	9	0	6	4	3	0	7	4	8	4	6	0
Eggerthellaceae	0	13	0	13	0	0	0	0	13	0	7	0	7	3	10	5	3	13	69	31
Adlercreutzia	29	53	71	53	41	31	42	71	53	41	29	26	41	34	90	25	20	53	52	50
Eggerthellaceae	26	46	29	46	33	9	13	29	46	33	19	25	31	16	58	24	24	19	19	23
Coriobacteriales	0	2	0	2	0	0	0	0	2	0	0	0	0	0	0	0	0	0	0	0
Senegalimassilia	52	48	50	48	47	23	20	50	48	47	46	21	41	32	77	28	31	53	29	29
Eggerthella	42	47	48	47	40	15	23	48	47	40	25	22	54	29	96	30	20	50	61	34
Slackia	0	2	4	2	0	6	5	4	2	0	4	3	0	0	4	8	9	0	0	3
Gordonibacter	29	27	18	27	18	12	13	18	27	18	11	11	27	15	63	14	16	42	153	65
Delftia	0	0	3	0	2	0	0	3	0	2	0	0	0	0	4	0	2	0	0	0
Parasutterella	37	49	51	49	36	21	33	51	49	36	29	30	68	58	141	19	18	79	61	70
Oxalobacter	6	11	10	11	9	0	9	10	11	9	11	11	12	16	21	10	0	12	12	14
Herbaspirillum	0	0	0	0	0	0	0	0	0	0	698	627	84	81	268	646	670	85	98	58
Burkholderia-Caballeronia	0	0	0	0	0	0	0	0	0	0	160	102	30	21	90	101	101	131	87	129
Oxalobacteraceae	0	0	0	0	2	0	0	0	0	2	0	0	0	0	0	0	0	0	0	0
vadinBE97	0	7	0	7	0	0	0	0	7	0	0	3	8	3	20	3	0	12	3	5
UCG-004	5	0	0	0	0	0	0	0	0	0	0	0	0	0	0	2	0	0	0	0
Erysipelatoclostridiaceae	0	0	5	0	0	0	2	5	0	0	2	0	0	0	0	0	0	0	0	0
Incertae_Sedis	318	290	309	290	185	128	177	309	290	185	224	179	170	88	363	162	135	227	150	163

Hits	No Substrate T0 n=2 T24 n=3					Fiber-Rich T0 n=2 T24 n=3					Western + BS T0 n=2 T24 n=3					Western - BS T0 n=2 T24 n=3				
	**n=1**	**n=2**	**n=1**	**n=2**	**n=3**	**n=1**	**n=2**	**n=1**	**n=2**	**n=3**	**n=1**	**n=2**	**n=1**	**n=2**	**n=3**	**n=1**	**n=2**	**n=1**	**n=2**	**n=3**
Caproiciproducens	0	5	7	5	0	0	0	7	5	0	0	0	0	0	0	0	0	0	0	0
Anaerotruncus	0	0	9	0	0	4	0	9	0	0	4	3	0	0	0	0	0	0	0	0
Negativibacillus	54	44	36	44	35	9	18	36	44	35	40	29	14	19	47	23	29	19	14	31
Phocea	11	7	4	7	4	4	4	4	7	4	0	7	0	0	4	8	5	0	6	0
DTU089	10	14	16	14	4	0	4	16	14	4	6	3	3	2	10	14	5	11	5	6
Oscillospirales	0	0	0	0	0	0	0	0	0	0	2	0	0	0	0	0	0	0	0	0
Anaerofilum	0	0	6	0	0	0	0	6	0	0	0	0	0	0	0	0	0	0	0	0
CAG-352	1067	1445	1388	1445	1091	487	620	1388	1445	1091	690	593	521	386	997	490	491	723	629	656
[*Eubacterium*]_*siraeum*	238	104	88	104	56	90	125	88	104	56	151	113	16	11	34	116	104	58	40	31

Supplementary statistical analysis (Fig. 2)

To quantitatively evaluate the compositional trends shown in Fig. 2, a statistical analysis (ΔT24-T0) was performed for all bacterial genera under each substrate condition to determine which composition most effectively preserved the original microbial community structure. For each genus, the condition with the smallest absolute deviation from baseline was identified, and group-level differences were statistically tested using the Kruskal-Wallis test. Among all tested conditions, the fiber-rich medium minimized compositional drift most reliably, being identified as the best-performing condition in 11 out of 34 genera, with statistical significance ($p < 0.05$) in 91 % of those cases. By contrast, the substrate-free condition showed the highest number of best-performer counts (14 genera), but with a notably lower significance rate (64 %), suggesting potential contributions from background effects or low selective pressure. The Western-style substrate conditions, with or without bile salts, yielded lower frequencies (5 and 7 genera, respectively), yet achieved statistical significance in all relevant comparisons, indicating condition-specific but strong compositional effects. These results collectively demonstrate that the fiber-rich formulation provided the most robust support for microbial stability and recovery in vitro, outperforming all other tested substrate compositions in both consistency and statistical reliability. In contrast, exposure to Western-style substrates induced shifts toward a dysbiotic community profile enriched in bile-tolerant and facultative anaerobic taxa.

Supplementary xenobiotic biotransformation datasets (Fig. 7)

To provide a parameter-based comparison of xenobiotic turnover across matrices, apparent first-order rate constants (k) and half-lives ($t_{1/2}$) were calculated from parent compound depletion data and are summarized in Supplementary Table S4. The kinetic evaluation confirmed the qualitative trends observed in the depletion plots: all compounds were essentially stable in buffer, whereas clear turnover occurred in fecal slurry and in the colon model. For roxatidine-acetate and chloramphenicol, kinetic parameters were nearly identical between fecal slurry and the colon model, while sulindac, sulfasalazine, and nitrendipine showed the same overall transformation pattern with minor quantitative differences. The exoenzyme fraction showed relevant activity only for ester hydrolysis substrates, particularly roxatidine-acetate and 4-acetoxyacetanilide, whereas the other compounds remained largely stable. These data support both the functional similarity between fecal slurry and the colon model and the mechanistic specificity of the system.

Table S4: Apparent first-order rate constants (*k*) and corresponding half-lives ($t_{1/2}$) for xenobiotic depletion in buffer, fecal slurry, colon model, and the corresponding exoenzyme fraction. Kinetic parameters were calculated from linear regression of ln (remaining precursor compound) versus time using quantifiable parent-compound concentrations only. Rate constants are given in h^{-1} and half-lives in h.

	Roxatidine-acetate		Sulindac		Sulfasalzine		Nitrendipine		4-Acetoxy-acetanilide		Chloramphe-nicol	
	k	$t_{1/2}$	*k*	$t_{1/2}$	*k*	$t_{1/2}$	*k*	$t_{1/2}$	*k*	$t_{1/2}$	*k*	$t_{1/2}$
**Buffer**	0.005	> 24	0.0003	> 24	0.002	> 24	0.0009	> 24	0.02	> 24	0.002	> 24
**Feces**	1.16	0.60	0.12	5.78	0.93	0.75	0.44	1.58	15.38	0.05	0.45	1.54
**Colon model**	1.13	0.61	0.20	3.47	1.20	0.58	0.92	0.75	21.18	0.03	0.47	1.47
**Exoenzymes**	0.16	4.33	0.003	> 24	0.003	> 24	0.002	> 24	13.46	0.05	0.003	> 24

Two additional xenobiotics representing distinct microbial reaction pathways are shown in Supplementary Figure S2. 4-Acetoxyacetanilide, used as a model substrate for ester hydrolysis, was rapidly degraded in fecal slurry and in the bacterial colon model, while the corresponding exoenzyme fraction also showed clear hydrolytic activity, consistent with an extracellular enzyme-driven reaction. In contrast, chloramphenicol, representing nitroreduction, was strongly transformed in fecal slurry and in the bacterial colon model, but remained essentially stable in the exoenzyme fraction, indicating that this reaction depended on intact bacterial cells rather than released extracellular enzymes. Overall, the degradation kinetics observed in fecal slurry and the colon model were highly comparable for both compounds, further supporting that the bacterial colon model preserves the relevant functional microbial activities while also allowing mechanistic discrimination between extracellular and cell-associated reactions.

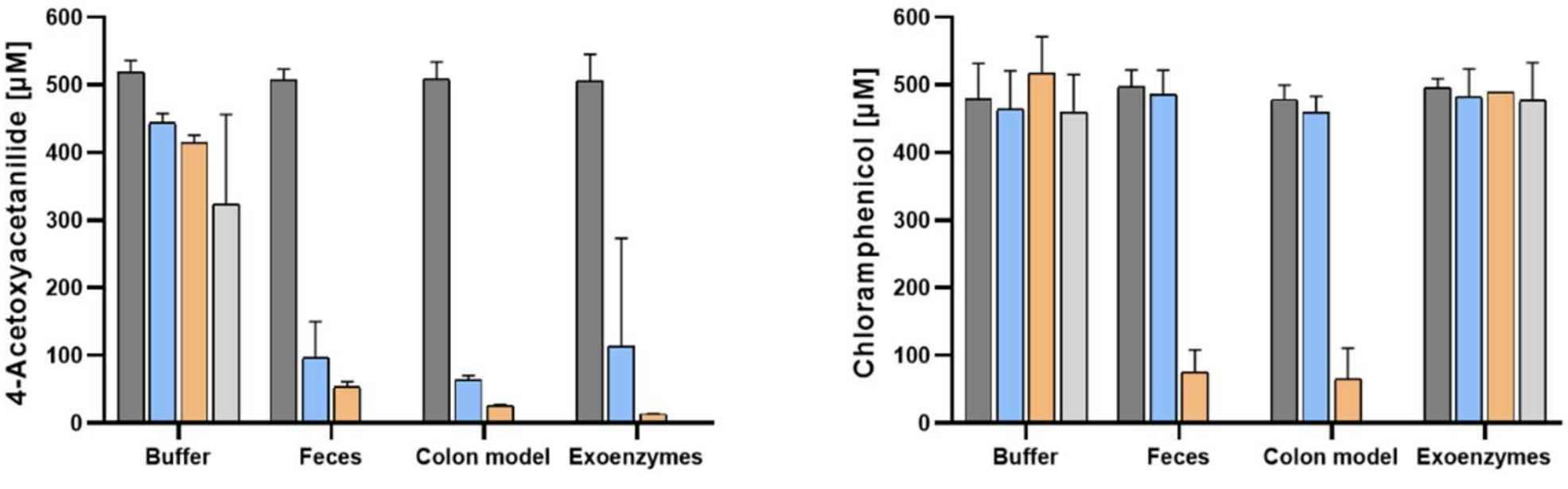

Fig. S2: Microbial biotransformation of xenobiotic model compounds in buffer, fecal slurry, colon model, and the corresponding exoenzyme fraction. Representative substrates were selected to cover different microbial transformation reactions commonly observed in the human colon: 4-acetoxyacetanilide for ester hydrolysis (A) and chloramphenicol for nitroreduction (B). Precursor-compound concentrations were quantified by HPLC at 0 h (dark grey), 0.1 h (blue), 4 h (orange), and 24 h (light grey) under anaerobic conditions (37°C). Data represent mean ± SD of three independent biological pools, each composed of three human donors.
